# Bioactivity of dihydropyrimidinone derivatives as inhibitors of cyclooxygenase-2 (COX-2): an *in silico* approach†

**DOI:** 10.1039/d3ra05942a

**Published:** 2023-11-23

**Authors:** Kautsar Ul Haq, Nur Lailatus Sa'adah, Imam Siswanto, Hery Suwito

**Affiliations:** a Bioinformatic Division, University CoE-Research Center for Bio-Molecule Engineering (BIOME), Airlangga University Surabaya 60115 Indonesia; b Department of Chemistry, Faculty of Science and Technology, Airlangga University Surabaya 60115 Indonesia hery-s@fst.unair.ac.id

## Abstract

Cyclooxygenase-2 (COX-2) is an enzyme involved in inflammation. The overexpression of COX-2 causes chronic inflammation, which can be prevented by COX-2 inhibitors. Generally, COX-2 inhibitors possess a carboxyl group and an aromatic ring in their molecular structure. These moieties are involved in the interaction with the active site of COX-2, thus playing a pivotal role in the inhibitory activity. Regarding the requisite molecular structure of COX-2 inhibitors, derivatives of dihydropyrimidinone (DHPM) are ideal candidates to be explored as COX-2 inhibitors, due to the ease of synthesis and their versatility to be transformed chemically. In this study, we prepared a novel small library consisting of 288 designed DHPM derivatives by varying the constituent components. The selection criteria of potential candidates for the COX-2 inhibitor of the data bank involve *in silico* studies *via* molecular docking investigations, prediction of ADMET and druglikeness, as well as molecular dynamics (MD) simulations. Molecular docking served as the initial step of selection, based on the comparison of grid score, docking pose, and interactions with those of lumiracoxib (LUR) as the original ligand of COX-2. The next criteria of selection were scores obtained from the ADMET and druglikeness by comparing the designed candidates with COX-2 inhibitors that were already marketed. Compound RDUE2 and SDT29 were the most potential candidates, which were further analyzed using the MD simulation. The results of the MD simulation indicated that RDUE2 and SDT29 interacted stably with amino acid residues on the active site of COX-2. The estimation of binding free energy indicated that SDT29 exhibited an inhibitory activity comparable to that of LUR, whereas RDUE2 showed a lower inhibitory activity than that of SDT29 and LUR.

## Abstract 2

Hundreds of dihydropyrimidinone derivatives were virtually screened to find novel COX-2-selective inhibitor candidates.

## Graphical abstract

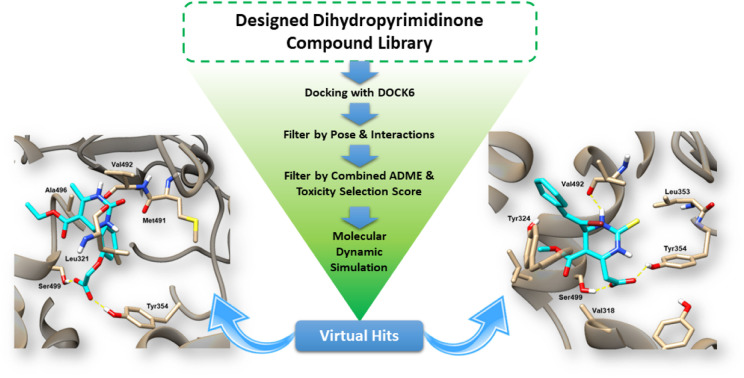

## Introduction

Inflammation is a response of the immune system to noxious stimuli, such as pathogens, damaged cells, toxic compounds, or irradiation,^1^ which is characterized by redness, swelling, heat, pain, and the loss of tissue function.^2^ This can become chronic if persistent for a long term, which can lead to tissue damage and cell death, causing various kinds of degenerative^3^ and neurodegenerative diseases.^4^ According to WHO, chronic inflammation is the most significant cause of death in the world.^5^ Diseases associated with chronic inflammation are expected to continue to increase over the next 30 years. Worldwide, 3 out of 5 people die from diseases associated with chronic inflammation, such as stroke, chronic respiratory disease, heart problems, cancer, obesity, and diabetes.^6–8^ Generally, anti-inflammatory drugs are COX-2 inhibitors, but these drugs cause unwanted side effects, such as the risk of heart and liver diseases.^9,10^ Therefore, new drug candidates for the treatment of inflammation are urgently needed.

The inhibition of COX-2 is the main solution in the treatment of inflammation. This enzyme is involved in the conversion of arachidonic acid to prostanoids, which are important substances involved in the occurrence of inflammatory processes.^11^ Therefore, COX-2 can be used as the target for the treatment of inflammation. One of the highly potent and selective drugs that inhibits COX-2 is lumiracoxib, which has an IC_50_ value of 0.13 μM with an excellent selectivity ratio (IC_50_ COX-1/IC_50_ COX-2) of 515 in the human whole blood assay.^12^ This drug has low gastrointestinal side effects;^13^ however, recently it has been reported to cause liver damage, leading to its withdrawal in 2007.^14,15^ Nevertheless, the molecular interaction between this drug and COX-2 serves as a good starting point for designing new inhibitors with different topologies to minimize the possibility of similar side effects. According to Carullo *et al.*,^16^ COX-2 inhibitors require two significant moieties that must be present in their structure, namely, carboxyl group and aromatic ring. These parts can bind with COX-2 to exhibit the desired inhibitory effect.

One of the compounds with diverse activities that can be easily designed according to the required structure is dihydropyrimidinone (DHPM).^17^ DHPM and their derivatives have been extensively studied and are known to have promising biological activities including anti-inflammatory,^18^ antioxidant,^19^ anticancer,^20^ and antimicrobial functions.^21^*In silico* studies proved that DHPM possesses the potential to act as a COX-2 inhibitor compared to Celecoxib.^17^ Alfayomy *et al.*^22^ revealed that DHPM shows a great IC_50_ value compared to Celecoxib. *In vivo* studies showed that DHPM has low side effects on gastrointestinal ulcers. Based on these findings, it is assumed that DHPM derivatives have great potential as COX-2 inhibitors.

This research was aimed to discover new COX-2 inhibitors through *in silico* studies. Candidates with DHPM were first designed as the main core, and then a docking experiment was performed to understand the inhibitor binding mode to the enzyme. ADMET prediction was used to study the drug-likeness of the candidates. MD simulations and binding free energy calculations were carried out to deeply investigate the interactions that occur between inhibitors and COX-2. This research is intended as a logical approach to the development and finding of potent inhibitors, showing good efficacy and efficiency as COX-2 inhibitors.

## Materials and methods

### Materials

COX-2 crystal complex with lumiracoxib (LUR) (PDB ID: 4OTY) was retrieved from RCSB Protein Data Bank,^23^ and 30 already marketed COX-2 inhibitors and 288 prior designed DHPM derivatives were used as candidates.

### Small molecular structure preparation

The 3D structure of COX-2 inhibitor candidates was constructed and assigned a protonation state at pH 7.4 using Avogadro.^24^ The structure was further optimized by a PM7 semi-empirical method implemented using Gaussian16.^25^ Finally, the addition of charges to the candidates was optimized by the AM1-BCC method using an antechamber program.^26^

### Molecular docking

A docking experiment was started with the validation of docking parameters that started by ligand and enzyme preparation using the DockPrep features in the Chimera program.^27^ The addition of charge to the receptor was calculated using the ff14SB method,^28^ whereas the non-protein part using the AM1-BCC method.^26^ The surface was made using the structure of a receptor that does not contain hydrogen atoms using the Write DMS feature in the Chimera program. Furthermore, the surface was used to create spheres using the SPHGEN program.^29^ The selection of spheres was done using the sphere selector program with a distance of 6.0 Å from the ligand position. After the spheres were selected, the simulation box was made using the SHOWBOX program with a radius of 7.0 Å.

The production of the grid was based on the Lennard-Jones model with an attractive and repulsion exponent of 6–9 using the GRID program.^30^ Finally, the redocking of the original ligand was carried out on the receptor by the flexible docking method using the DOCK6 program.^31^ The pose reproduction was considered successful if the redocking results show the same ligand pose as the original ligand before the docking process and have an RMSD value of ≤2.0 Å.^32^ Then all the ligand candidates were docked to COX-2, and the selection of candidates for further analyses is based on the grid score and poses produced during the docking process.

### ADMET analysis

The ADMET properties were predicted using the SwissADME web service^33^ and Toxicity Estimation Software Tool (T.E.S.T) program,^34^ and the analysis was performed for 30 marketed COX-2 inhibitors and 288 candidates. Candidate selection criteria were assessed using the selection score (SS),^35^ which includes the ADMET parameter and grid score from docking. Candidates that showed a higher SS than the reference (ibuprofen) then proceeded to the MD simulation.

### Molecular dynamics simulation

Molecular dynamics (MD) simulation was used to study protein–inhibitor interactions.^36^ The used ligands and receptors were retrieved from the previously docking process. All simulations were carried out using the AMBER22 program.^37^ Protein was processed under the ff14SB force field,^28^ and inhibitors were processed under the GAFF force field.^38^ The complex in the solution phase was modelled using the TIP3PBOX water model with a radius of 10 Å. A total of four Na^+^ ions were added to neutralize the protein–ligand complex system.

The system minimization was carried out by the steepest descent and conjugate gradient method. The minimization stage consists of three steps using the sander program in AMBER22: (i) minimization of water molecules as a solvent with a force constant of 500 kcal mol^−1^ Å^−1^ and hold on the N and C terminal protein (3000 cycles of steepest descent and 2000 cycles of conjugate gradient); (ii) minimization of water molecules and side chain atoms of the complex with a force constant of 500 kcal mol^−1^ Å^−1^ and hold on residues on the active side and at the end of the protein (1000 cycles of steepest descent and 2000 cycles of conjugate gradient); and (iii) finally, the minimization of all atoms in the system of 2000 cycles without any restrictions (1000 cycles of steepest descent and 1000 cycles of conjugate gradient).

The heating process was carried out for 200 ps from 0 K to 310 K on the NVT Ensemble. Adjustment of the system density was carried out for 300 ps at 310 K. The equilibration system was carried out with ten consecutive stages using partial restraints of 20.0, 10.0, 5.0, 2.5, 1.0, 0.5, 0.1, 0.05, and 0.001, and without restraints at all; in the residues on the active site, as well as C and N terminals of enzyme for 4500 ps on the NPT ensemble. Then, the MD simulation was carried out for 150 ns to find out the stability of the system during the simulation process. The coordinates are stored every 5 ps. All simulations were carried out using the sander program in AMBER22.

### Binding free energy calculation

Binding free energy calculation was used to study the binding affinity of inhibitors on the protein. To calculate the binding free energy, 2000 snapshots obtained from the last 10 ns were used. Molecular Mechanics-Poisson Boltzmann Surface Area (MM-PBSA) and Molecular Mechanics-Generalized Born Surface Area (MM-GBSA) were used in the calculation of binding free energy.^39^ Thus, the binding free energy (Δ*G*_bind_) between inhibitors (lig) and COX-2 (rec) was calculated using the following equation:

1 Δ*G*_bind_ = *G*_com_ − (*G*_rec_ + *G*_lig_)

2 Δ*G*_bind_ = Δ*H* − *T*Δ*S* ≈ Δ*E*_MM_ + Δ*G*_sol_ − *T*Δ*S*

3 Δ*E*_MM_ = Δ*E*_int_ + Δ*E*_ele_ + Δ*E*_vdw_

4 *G*_sol_ = Δ*G*_GB/PB_ + Δ*G*_SA_

where *G*_com_, *G*_rec_, and *G*_lig_ are the free energy from complexes, receptors, and ligands, respectively. Δ*G*_bind_ gives the gas phase enthalpy (Δ*E*_MM_), solvation-free energy (Δ*G*_sol_), and conformational entropy (−*T*Δ*S*) in the binding of ligands. Δ*E*_MM_ is the amount of internal energy/Δ*E*_int_ (bond, angle, and dihedral energies), electrostatic interactions/Δ*E*_ele_, and van der Waals interactions/Δ*E*_vdw_. Δ*G*_sol_ is the number of polar contributions (Δ*G*_GB/PB_) and nonpolar contributions (Δ*G*_SA_).

The polar contribution was calculated by the PB and GB approach using programs available in AMBER22.^37^ We used 80 for the exterior dielectric constant and 1 for the solute dielectric constant. The nonpolar part of desolvation was estimated from the solvent accessible surface area (SASA) using the LCPO method with a water probe radius of 1.4 Å: Δ*G*_SA_ = 0.0072 × ΔSASA. Here, changes in conformational entropy −*T*Δ*S* are not considered due to expensive computational costs and low prediction accuracy.^40^

### Trajectory analysis

Detailed information on protein–ligand binding was obtained by studying the contribution of each amino acid residue to the interaction energy between the inhibitor and COX-2 using the MM-PBSA and MM-GBSA methods in AMBER22.^41^ The binding contribution of each residue includes four things: van der Waals (Δ*E*_vdw_), electrostatic (Δ*E*_ele_), polar solvation (Δ*G*_GB/PB_), and nonpolar solvation (Δ*G*_SA_), without considering the entropy contribution.

In addition, trajectory analysis also includes RMSF, RoG, and SASA analyses to study the stability of each system.^42^ Trajectory analysis was performed at 2000 frames from the last 10 ns of the MD trajectory.

## Results and discussion

### Design of inhibitors

Generally, COX-2 inhibitors possess a carboxyl group and an aromatic ring in their structure.^11,43^ The carboxyl group of COX-2 inhibitors interacts with the enzyme through hydrogen bonds, while the aromatic ring interacts at the non-polar part of the COX-2 active site. Such structure is found in many anti-inflammatory drugs, such as lumiracoxib, diclofenac, etodolac, flurbiprofen, and ketoprofen.^44^

DHPM is a class of compounds that are easy to be synthesized and chemically transformed to meet the structural requirements needed for COX-2 inhibitors. The Biginelli reaction is usually used in the synthesis of DHPM derivatives by mixing the reaction components, namely, derivatives of aldehyde (A), 1,3-dicarbonyl (B), and urea (C) in a reaction vessel.^45^ For the aldehyde component, several substituted benzaldehydes, heteroaromatics, and cinnamaldehyde derivatives were used. In the carbonyl component, we used 3 aliphatic 1,3-diketones. For the urea component, simple urea and cyclic urea derivatives were employed. A small library was created by enumerating these three components, resulting in a total of 288 compounds, including their stereoisomers. The first letter of the compound name refers to absolute configuration information (ESI, Table S1†). The core structure of the candidates is displayed in Table 1.

**Table 1:** Component variations to make DHPM: (A) benzaldehyde, (B) 1,3-dicarbonyl, (C) urea/thiourea; and the main core of the candidates

Component variations	Main core of candidates
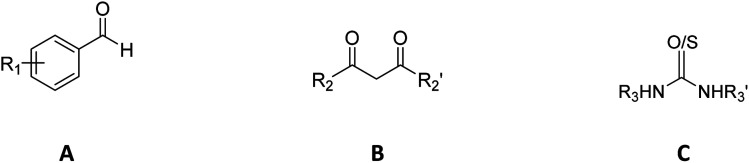	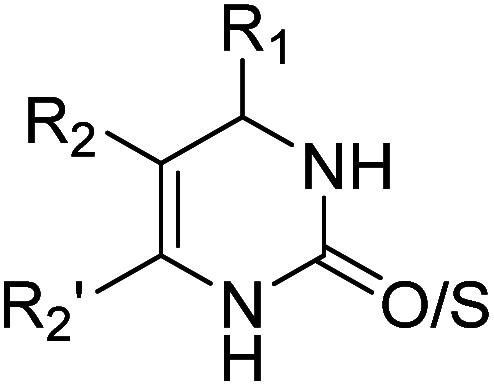

### Docking analysis

**Fig. 1:**
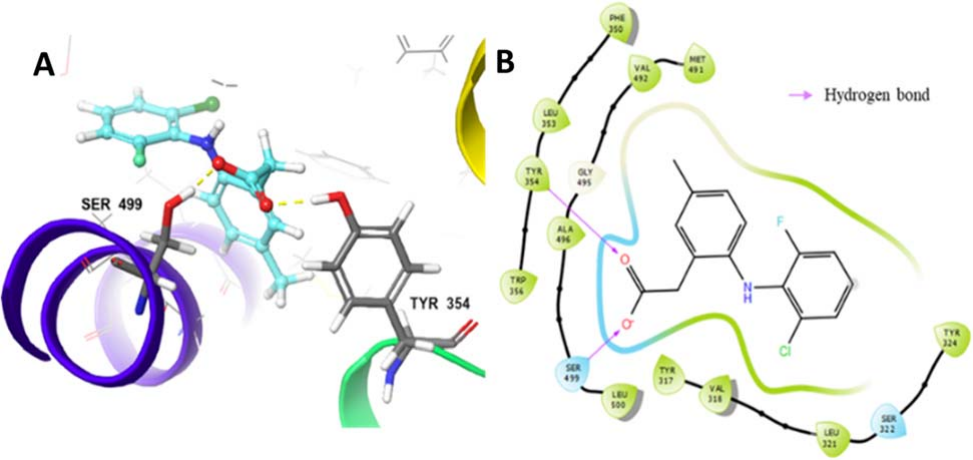
Interaction between LUR and COX-2 based on molecular docking: (A) 3D visualization and (B) 2D visualization.

Docking was used to study the interaction mode of the inhibitor on COX-2. The docking of the original ligand, lumiracoxib (LUR), gave a grid score of −64.16 kcal mol^−1^. The carboxyl group of LUR built two hydrogen bonds with Tyr354 and Ser499. The 3D and 2D visualization of the interaction between LUR and COX-2 is presented in Fig. 1.

Then, we docked 288 designed candidates and 30 marketed COX-2 inhibitors. The information obtained from the results comprised grid score, docking pose, and interaction of candidates with COX-2. To maximize prediction accuracy, the docking poses of candidates should overlap with the cognate ligand (LUR) and the candidates with similar interaction modes to LUR are expected to show better or at least equivalent activity to LUR.^46,47^ The results showed that only one candidate had a lower grid score than that of LUR. However, its docking pose did not have same interaction and did not overlap with LUR, so it was not selected for further analyses.

The docking results of LUR and 14 selected candidates are shown in Table 2. Generally, the carboxyl group of the candidates forms hydrogen bonds with residues Tyr354 and Ser499. Based on these data, it is known that DHPM synthesized from the aldehyde component with a phenoxyacetate moiety predominantly occupies the top rank. The selection process was then followed by the ADMET and druglikeness prediction.

**Table 2:** Docking results of LUR and 14 selected candidates

No.	Ligand	Gridscore (kcal mol^−1^)	Type of interaction	Amino acid residue
	LUR	−64.16	Hydrogen bond	Tyr354; Ser499
1	RDUE2	−63.96	Hydrogen bond	Tyr354; Ser499
2	SDTE3	−62.17	Hydrogen bond	Tyr324; Ser499
3	RDUA2	−61.20	Hydrogen bond	Tyr354; Ser499
4	RDTE2	−58.66	Hydrogen bond	Tyr354; Ser499
5	SDUA2	−57.60	Hydrogen bond	Tyr354; Ser499
6	SDZA2	−55.34	Hydrogen bond	Tyr354; Ser499
7	SDTA2	−54.04	Hydrogen bond	Tyr354; Ser499
8	SDT25	−52.68	Hydrogen bond	Tyr354; Ser499
9	RDTA2	−51.33	Hydrogen bond	Tyr354; Ser499
10	RDZE2	−48.24	Hydrogen bond	Tyr324; Tyr354; Ser499
11	SDT29	−47.80	Hydrogen bond	Tyr354; Met491; Ser499
12	RDZA2	−47.79	Hydrogen bond	Tyr324; Ser499
13	SDU29	−47.32	Hydrogen bond	Tyr354; Ser499
14	SDT28	−39.41	Hydrogen bond	Tyr354; Ser499

### Selection of candidates

**Fig. 2:**
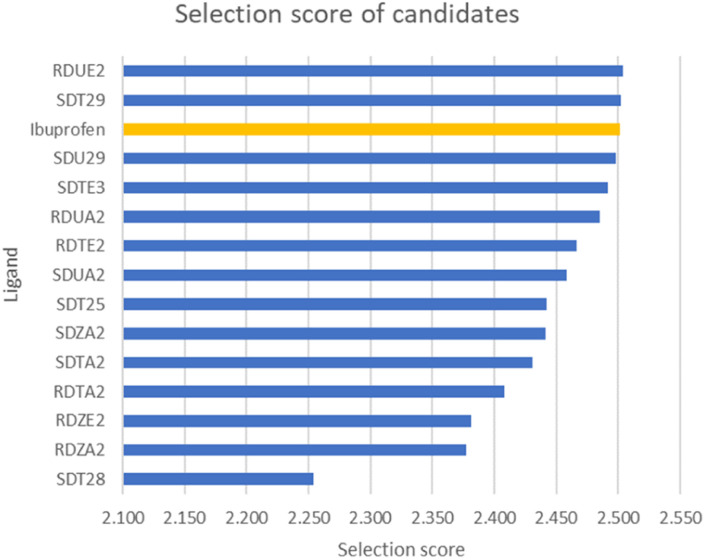
Graph of SS analysis of the selected candidates and ibuprofen.

The selection of candidates was based on ADMET and docking parameters. Based on these parameters, Castro-González *et al.*^35^ created an assessment model called the selection score (SS) to sort drug candidates by comparing the parameter scores of the candidate with the parameter score of ibuprofen as drug references. Ibuprofen was used as a reference because it is well known for its activity as an COX-2 inhibitor and in general has been used by the public as an anti-inflammatory drug.^48^ The results of the selection score analysis of 14 selected candidates are displayed in Fig. 2.

**Fig. 3:**
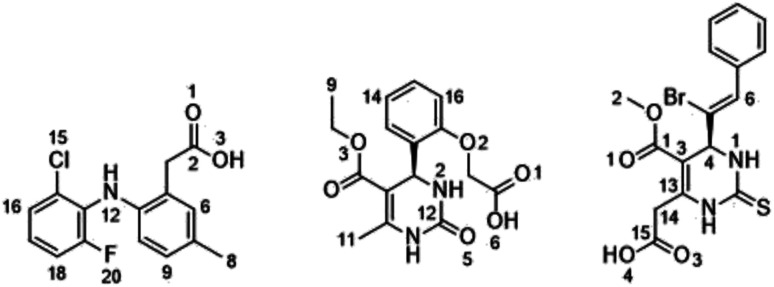
Atomic numbering in the ligand: (A) LUR, (B) RDUE2, and (C) SDT29.

Among 10 candidates, only two candidates exhibited higher SS values than ibuprofen, namely, (*R*)-2-(2-(5-(ethoxycarbonyl)-6-methyl-2-oxo-1,2,3,4-tetrahydropyrimidin-4-yl)phenoxy)acetate (RDUE2) and (*S*,*Z*)-2-(6-(1-bromo-2-phenylvinyl)-5-(methoxycarbonyl)-2-thioxo-1,2,3,6-tetra-hydropyrimidin-4-yl)acetate (SDT29). Both candidates are expected to show ADMET and drug-likeness properties comparable to or even better than marketed drugs, so that they can serve as promising drug candidates. The structure and numbering of the two selected candidates are presented in Fig. 3.

### Molecular dynamics simulation

#### Structural flexibility and stability

In general, molecular dynamics simulation was carried out on a protein–ligand system to explore its stability and energy. The MD simulation was run for 150 ns on both the apo-COX-2 system and the protein–ligand system (COX-2:LUR, COX-2:RDUE2, and COX-2:SDT29). The complex stability was assessed based on the root mean square deviation (RMSD) of the atoms in the backbone and ligands.

**Fig. 4:**
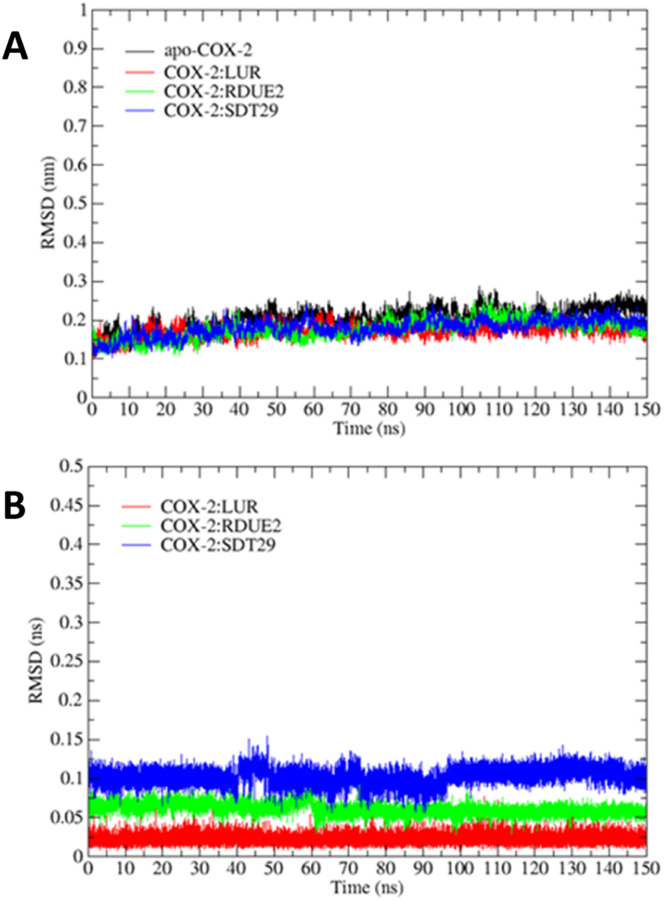
RMSD of each system for 150 ns: (A) backbone and (B) ligand.

Fig. 4 describes the RMSD stability of the backbone and ligand during simulation, and it was achieved during the last 10 ns of the simulation, ranging from 0.17 to 0.24 nm and 0.02 to 0.10 nm, respectively. The apo-COX-2 system showed the largest RMSD backbone, which indicated that the free COX-2 structure tends to have a greater structural change than that in a complex system.

**Fig. 5:**
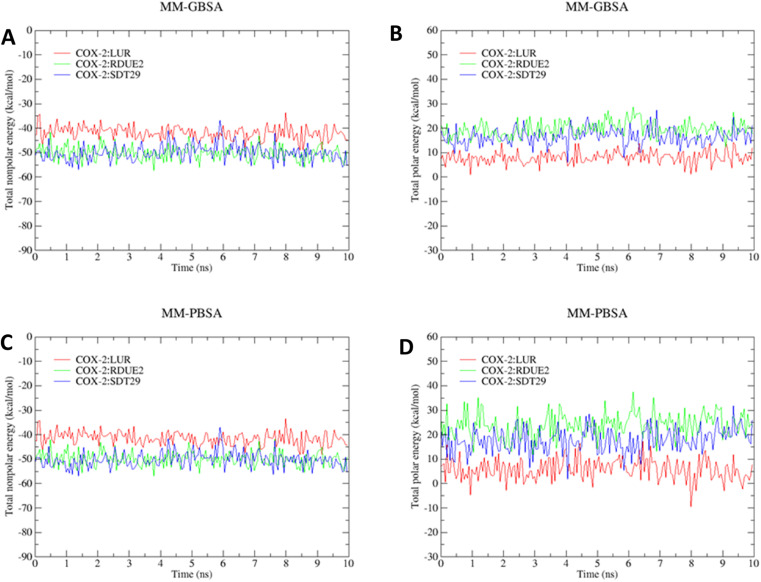
Stability of (A) total nonpolar energy (Δ*E*_vdw_ + Δ*G*_SA_), (B) total polar energy (Δ*E*_ele_ + Δ*G*_GB_) based on the MM-GBSA approach, (C) total nonpolar energy (Δ*E*_vdw_ + Δ*G*_SA_), and (D) total polar energy (Δ*E*_ele_ + Δ*G*_PB_) based on the MM-PBSA approach.

However, the parameters of stability were determined not only by RMSD value but also by free energy. Therefore, the next parameter of complex stability to be determined was energy stability during simulation. This parameter was obtained from the MM-GBSA and MM-PBSA approach, including the energy of non-polar (van der Waals) and polar interactions (electrostatic interaction and hydrogen bonding), as shown in Fig. 5.

The total (nonpolar and polar) energy analyzed using MM-GBSA and MM-PBSA approaches was stable, which declared that all systems were relatively stable, and they did not require an additional longer time of simulation. In addition, all three systems achieved their stability at about the same time.

The achievement of stability as shown in the analysis of RMSD and total energy was then continued with trajectory analysis, which was carried out for the last 10 ns of the simulation (140–150 ns). It comprises root mean square fluctuation (RMSF), radius of gyration (RoG), and solvent accessible surface area (SASA).

**Fig. 6:**
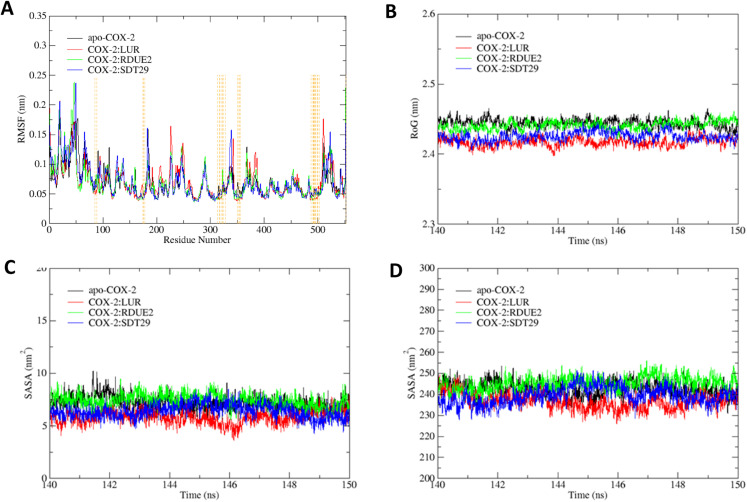
Trajectory analysis of each system: (A) RMSF, (B) RoG, (C) SASA on the active site, and (D) SASA on the entire enzyme.

RMSF analysis was used to determine the system flexibility through fluctuations of amino acid residues.^49^Fig. 6A shows that all systems fluctuate at residues of 1–2, 18–22, 41–53, 66–67, and 552. Specifically, residue numbers 183–184 fluctuate in apo-COX-2 and COX-2: SDT29. Several fluctuations only occur in COX-2: LUR, namely, residues 227, 343, and 511, while fluctuations in residue numbers 339 and 523 only occur in COX-2:SDT29. Overall, the fluctuations that occurred in each system are residues that are quite far from the active site of COX-2. This observation gave us information that the active site of the enzyme has low flexibility, which allows for a more stable interaction between ligands and COX-2.

The compactness of systems was assessed by the RoG of each system, as shown in Fig. 6B. It shows that each system has relatively the same RoG but with a quite small deviation, so that it can be assumed that the entire system is stable and compact. Fig. 6B shows that the RoG of the system from smallest to largest is: COX-2:LUR, COX-2:SDT29, COX-2: RDUE2, and apo-COX-2. According to Khan *et al.*,^50^ the lower the RoG of a system, the more compact the system is. The system with a ligand on the active site of COX-2 possessed a lower RoG than the apo system. This proves that ligands that interact with COX-2 can affect the structure and compactness of the overall structure of the enzyme.

Solvent accessible surface area (SASA) is one of the determining factors in studying the folding and stability of enzyme structures.^51^ Enzyme structures that experience an unfolded state tend to have a higher SASA than those with stably folded structures.^52^ In this study, SASA analysis was performed on the active site and the entire enzyme. The results indicated that for both the active site and the whole enzyme, apo-COX-2 had the highest SASA, while COX-2:LUR provided the smallest SASA. This indicates that the presence of a ligand on the active site of COX-2 results in a more stable complex structure than the structure of the enzyme without any ligand. The presence of candidates certainly also affects the stability of the folding of COX-2, which can be seen by its SASA. The average SASA of each system is shown in Fig. 6C and D.

#### Binding free energy calculation

The stability and affinity of each complex are represented by the binding free energy (Δ*G*_bind_).^53^ MM-PBSA and MM-GBSA were used to estimate Δ*G*_bind_ when ligands interact with COX-2.^39^ The last 10 ns trajectory of the simulation was used to calculate Δ*G*_bind_, under consideration that the stability of each system had been achieved. It means that there are no significant fluctuations in this trajectory, so that it is useful for increasing the efficiency of calculation when conducting analysis.^42^ Δ*G*_bind_ for each system is shown in Table 3.

**Table 3:** Prediction of binding free energy (kcal mol^−1^ ± average standard error) using MM-PBSA and MM-GBSA

Component of energy	COX-2:LUR	COX-2:RDUE2	COX-2:SDT29
Δ*E*_vdW_	−36.542 ± 0.185	−44.053 ± 0.200	−44.092 ± 0.217
Δ*E*_ele_	−42.044 ± 0.488	−30.286 ± 0.710	−48.408 ± 0.535
Δ*G*^ele^_GB_	50.039 ± 0.432	49.963 ± 0.588	64.917 ± 0.480
Δ*G*^solv^_GB_	44.701 ± 0.431	43.840 ± 0.586	58.964 ± 0.478
Δ*G*^bind^_GB_	−33.888 ± 0.160	−30.498 ± 0.243	−33.536 ± 0.242
Δ*G*^ele^_PB_	47.850 ± 0.495	54.224 ± 0.653	66.261 ± 0.477
Δ*G*^solv^_PB_	42.879 ± 0.497	48.318 ± 0.654	60.171 ± 0.477
Δ*G*^bind^_PB_	−35.710 ± 0.277	−26.021 ± 0.323	−32.329 ± 0.317

The results revealed that COX-2:RDUE has the largest Δ*G*_bind_ value compared to others. While COX-2:SDT29 exhibits a similar Δ*G*_bind_ value to COX-2:LUR used as a reference. This indicated that SDT29 has an activity equivalent to LUR and better than RDUE2.

#### Analysis of energy decomposition

**Fig. 7:**
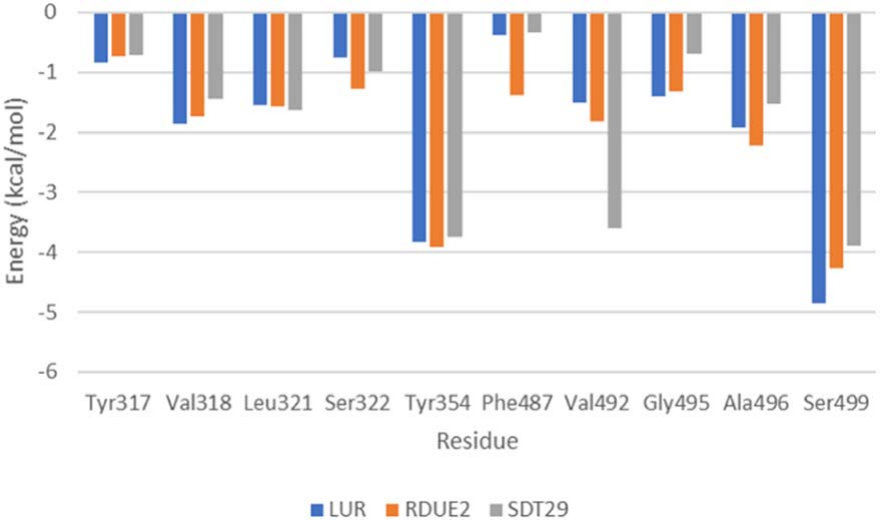
Energy decomposition analysis of residues which greatly contribute to the stability of the complex.

Energy decomposition analysis was used to find out which contributions are given by residues for the stability of the ligand–enzyme complex. Based on the results, only a small portion of residues greatly contribute to binding free energy. In addition, there are no residues that contribute to unfavorable interactions such as repulsion between ligands and enzymes. The graph of energy decomposition analysis is shown in Fig. 7.

**Fig. 8:**
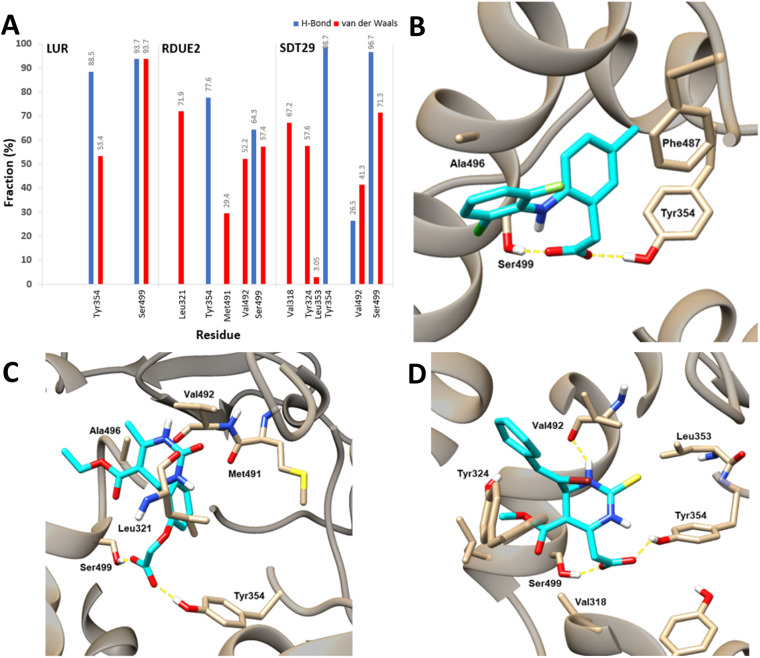
Contribution for each residue based on the decomposition energy for COX-2 (A) and residue interactions with (B) LUR, (C) RDUE2, and (D) SDT29.

Energy decomposition analysis shows that Tyr354 and Ser499 are residues that gave the greatest contribution to the binding free energy of the three complexes. However, in COX-2:SDT29, Val492 also gave a significant contribution to the stability of the complex. The presence of this additional contribution causes COX-2:SDT29 to have good stability and a lower binding free energy compared to COX-2:RDUE2. It is also confirmed that SDT29 inhibition activity towards COX-2 is better and comparable to LUR than RDUE2. The analysis of the type of contribution for each residue based on decomposition energy is shown in Fig. 8A and Tables S4, S5.†

It is shown that Tyr354 and Ser499 are two beneficial residues for the stability of the three complexes, by contributing to electrostatic energy. This is in line with the results of molecular docking analysis, where ligands form hydrogen bonds with COX-2 through these two residues. However, the energy of the polar solvation of these residues is unfavorable for the stability of the complex. In the complex of COX-2:LUR and COX-2:RDUE2, Val492 provided electrostatic energy that is not beneficial for complex stability. However, Val492 shows beneficial electrostatic energy in COX-2:SDT29.

In addition to the electrostatic energy, several other residues make a quite good contribution to the stability of the complex through the van der Waals interaction. The residues include Tyr317, Val318, Leu321, Ser322, Phe487, Val492, Gly495 and Ala496. These residues interact with non-polar parts of ligands, such as aromatic rings in LUR, RDUE2, and SDT29.

#### Residue interaction

The interaction of ligands with residues around the active side of COX-2 was studied with contact analysis performed on the last 10 ns trajectory. The contact analysis process was focused on amino acid residues with a radius of 3.5 Å from the active side of the enzyme. In general, there are two types of interactions between ligands and residues on each complex, namely, hydrogen bonds and van der Waals interactions. Contact analysis is focused on the percentage of interactions possessing a fraction >1%. The fraction can be calculated using eqn (5), which shows the percentage of interaction (fraction) of the number of frames for each interaction (*N*_fra_) divided by the total number of frames (*N*_tot_) during the simulation of 2000 frames.^54^5
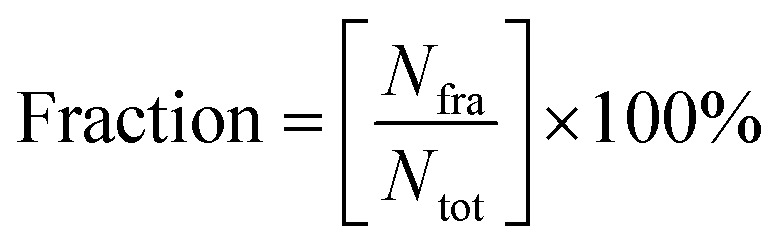


Hydrogen bond interaction between residues and ligands showed high fraction and was assumed to contribute most of the complex stability in each system. Fig. 8A shows that during the last 10 ns of the simulation time, on average there are two hydrogen bonds that occur in the COX-2:LUR and COX-2:RDUE2 systems. However, the occurrence of hydrogen bonds in the COX-2:LUR system is considered more consistent than that in the COX-2:RDUE2. However, on average, there are three bonds that occur in the COX-2:SDT29 system. This shows that the stability of COX-2:SDT29 is better than that of COX-2:RDUE2. This data showed that Ser499 and Tyr354 are the two main residues playing a major role in hydrogen bonds in each complex. Besides both interactions, in complex COX-2:SDT29, an additional hydrogen bond interaction with Val492 in lower fractions was observed.

In addition to the hydrogen bonds, complex stability was supported by van der Waals interactions between ligands and COX-2, as shown in Fig. 8A. Contact analysis informed that LUR, RDUE2, and SDT29 also interact with COX-2 *via* van der Waals interactions through Ser499 in high fractions. Besides Ser499, RDUE2 shows van der Waals interactions in high fractions with Leu321, while SDT29 shows van der Waals interactions with Val318 and Tyr324. In addition to the interaction of van der Waals in high fractions, there are quite several other van der Waals interactions in lower fractions, which were expected to support the stability of the complex. Briefly, all these observed interactions between ligands and receptors supported the inhibitory activity against COX-2.

## Conclusions

A series of DHPM derivatives have been designed as COX-2 inhibitors. Their inhibitory activities were deduced from the results of molecular docking, ADMET prediction, drug-likeness, and molecular dynamics simulation. The selection of the potential candidates was based on the criteria reported by Castro-González *et al.*^35^ The structure of COX-2 in the complex with lumiracoxib was retrieved from the Protein Data Bank (PDB ID: 4OTY), while 288 candidates were designed of dihydropyrimidinone scaffolds as products of the Biginelli reaction, and 30 drugs known as COX-2 inhibitors were used as reference in drug-likeness analysis. Thirty two of 288 candidates showed better grid score and similar binding mode to LUR, which were then taken for the selection of drug-likeness, which comprised pharmacokinetics and toxicity. The selection scores obtained were then used to determine the potential candidates. Based on the *in silico* study, two candidates, namely, RDUE2 and SDT29 are predicted to possess potential inhibitory activities, which are comparable to LUR. Amino acid residues playing a pivotal role in the inhibition activity are Tyr354 and Ser499 *via* the formation of hydrogen bonding with the carboxyl group of the ligand.

For molecular dynamics simulations, apo-COX-2, COX-2:LUR, COX-2:RDUE2, and COX-2:SDT29 systems were used to study the stability and energy of each system. The estimation of binding free energy, energy decomposition analysis, and interaction analysis between residues and ligands indicate that RDUE2 and SDT29 have promising potential as COX-2 inhibitors compared to LUR. The binding free energy of SDT29 was smaller, and the residue number of Ser499, Tyr354, and Val492 contributed to the stability of COX-2:SDT29. However, the binding free energy of COX-2:RDUE2 was greater, and only Ser499 and Tyr354 contribute to their stability. The energy decomposition analysis showed that the main residue contributes to complex stability *via* electrostatic interaction. The results indicated that the hydrogen bonds between candidates and COX-2 are very useful for increasing the inhibitory activity. These results are following our assumptions when designing new COX-2 inhibitors based on DHPM, and the carboxyl group is an important part because it endows the ligands with the ability to interact with COX-2 through hydrogen bonds, causing the expected inhibitory activities.

## Author contributions

K. U. H.: conceptualization, formal analysis, investigation, methodology, writing – review; N. L. S.: formal analysis, investigation, visualization, writing – original draft; I. S.: validation, writing – review and editing; H. S.: project administration, supervision, writing – review and editing.

## Conflicts of interest

There are no conflicts to declare.

## Supplementary Material

RA-013-D3RA05942A-s001

RA-013-D3RA05942A-s002
