# Supplementary material for: Bioactivity of dihydropyrimidinone derivatives as inhibitors of cyclooxygenase-2 (COX-2): an in silico approach
Source: RSC Adv. 2023 Nov 23;13(49):34348–57. doi: 10.1039/d3ra05942a (PMC10665647; doi:10.1039/d3ra05942a)
Supplement: RA-013-D3RA05942A-s002 [file RA-013-D3RA05942A-s002.pdf]

Electronic Supplementary Material (ESI) for RSC Advances.
This journal is © The Royal Society of Chemistry 2023

Supplementary Materials

Bioactivity of dihydropyrimidinone derivatives as inhibitors of cyclooxygenase-2 (COX-2): an in silico approach

Kautsar Ul Haq[a,b], Nur Lailatus Sa'adah[b], Imam Siswanto[a,b], and Hery Suwito[b*]

[a.] *Bioinformatic Division, University CoE-Research Center for Bio-Molecule Engineering (BIOME), Airlangga University, Surabaya 60115, Indonesia*
[b.] *Department of Chemistry, Faculty of Science and Technology, Airlangga University, Surabaya 60115, Indonesia*

Table S1. List of designed candidates with DHPM core

Code	Structure	Code	Structure
DB01	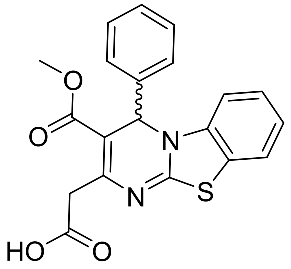	DU13	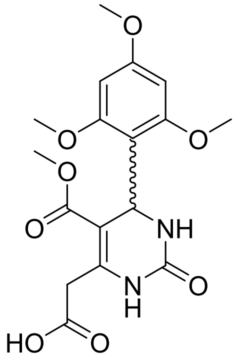
	O=C(O)CC1=C(C(C2=CC=CC=C2)N3C4=C(C=CC=C4)SC3=N1)C(OC)=O		O=C(O)CC1=C(C(C2=C(OC)C=C(OC)C=C2OC)NC(N1)=O)C(OC)=O
DB02	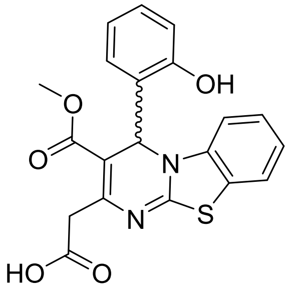	DU14	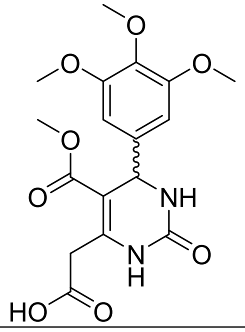
	O=C(O)CC1=C(C(C2=C(O)C=CC=C2)N3C4=C(C=CC=C4)SC3=N1)C(OC)=O		O=C(O)CC1=C(C(C2=CC(OC)=C(OC)C(OC)=C2)NC(N1)=O)C(OC)=O
DB03	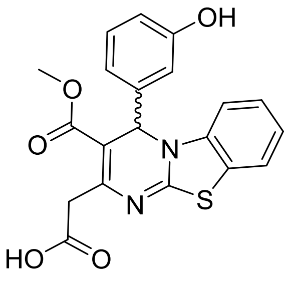	DU15	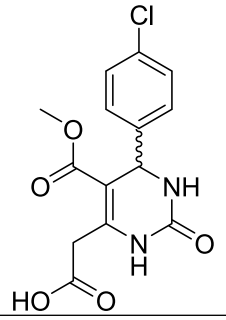
	O=C(O)CC1=C(C(C2=CC(O)=CC=C2)N3C4=C(C=CC=C4)SC3=N1)C(OC)=O		O=C(O)CC1=C(C(C2=CC=C(Cl)C=C2)NC(N1)=O)C(OC)=O

DB04	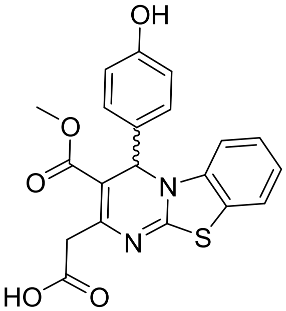	DU16	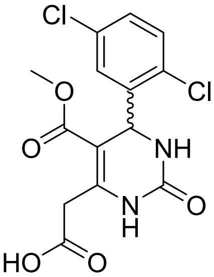
	O=C(O)CC1=C(C(C2=CC=C(O)C=C2)N3C4=C(C=CC=C4)SC3=N1)C(OC)=O		O=C(O)CC1=C(C(C2=C(Cl)C=CC(Cl)=C2)NC(N1)=O)C(OC)=O
DB05	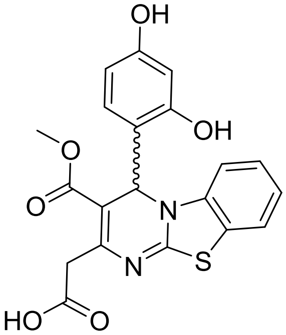	DU17	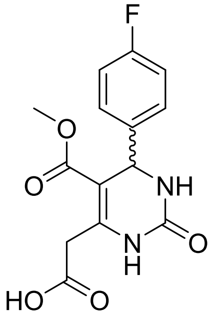
	O=C(O)CC1=C(C(C2=C(O)C=C(O)C=C2)N3C4=C(C=CC=C4)SC3=N1)C(OC)=O		O=C(O)CC1=C(C(C2=CC=C(F)C=C2)NC(N1)=O)C(OC)=O
DB06	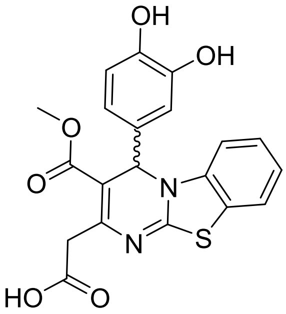	DU18	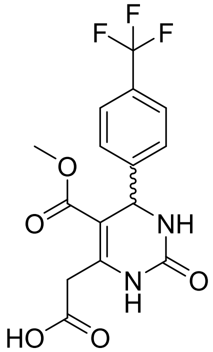
	O=C(O)CC1=C(C(C2=CC(O)=C(O)C=C2)N3C4=C(C=CC=C4)SC3=N1)C(OC)=O		O=C(O)CC1=C(C(C2=CC=C(C(F)(F)F)C=C2)NC(N1)=O)C(OC)=O
DB07	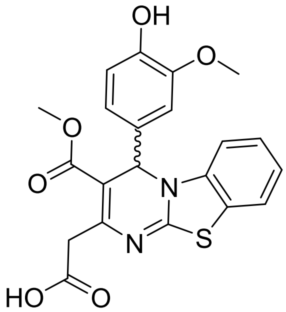	DU19	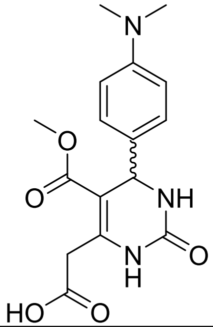
	O=C(O)CC1=C(C(C2=CC(OC)=C(O)C=C2)N3C4=C(C=CC=C4)SC3=N1)C(OC)=O		O=C(O)CC1=C(C(C2=CC=C(N(C)C)C=C2)NC(N1)=O)C(OC)=O

DB08	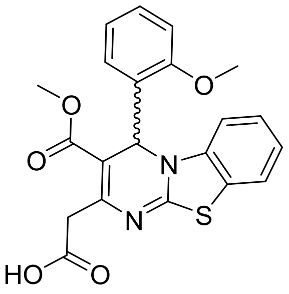	DU20	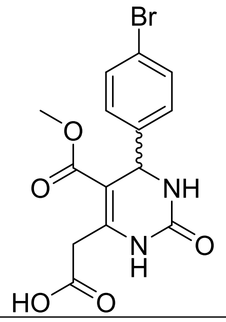
	O=C(O)CC1=C(C(C2=C(OC)C=CC=C2)N3C4=C(C=CC=C4)SC3=N1)C(OC)=O		O=C(O)CC1=C(C(C2=CC=C(Br)C=C2)NC(N1)=O)C(OC)=O
DB09	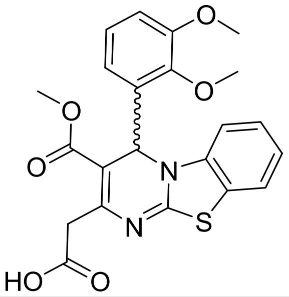	DU21	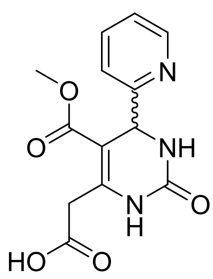
	O=C(O)CC1=C(C(C2=C(OC)C(OC)=CC=C2)N3C4=C(C=CC=C4)SC3=N1)C(OC)=O		O=C(O)CC1=C(C(C2=NC=CC=C2)NC(N1)=O)C(OC)=O
DB10	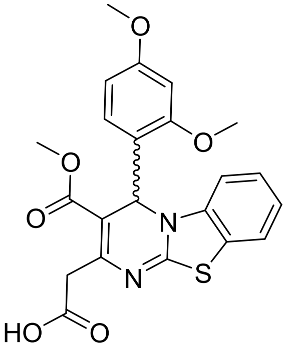	DU22	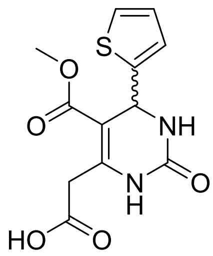
	O=C(O)CC1=C(C(C2=C(OC)C=C(OC)C=C2)N3C4=C(C=CC=C4)SC3=N1)C(OC)=O		O=C(O)CC1=C(C(C2=CC=CS2)NC(N1)=O)C(OC)=O
DB11	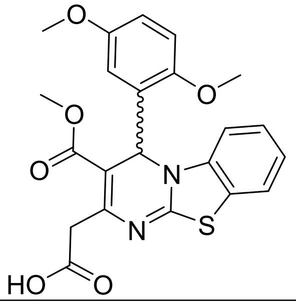	DU23	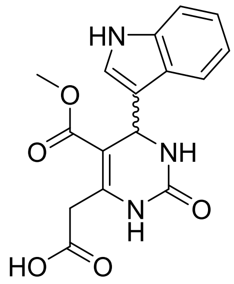
	O=C(O)CC1=C(C(C2=C(OC)C=CC(OC)=C2)N3C4=C(C=CC=C4)SC3=N1)C(OC)=O		O=C(O)CC1=C(C(C2=CNC3=C2C=CC=C3)NC(N1)=O)C(OC)=O

DB12	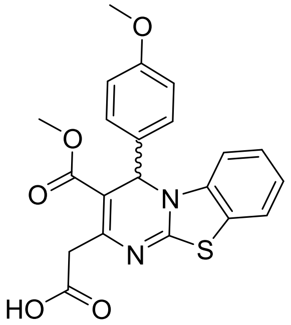	DU24	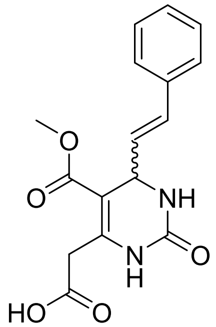
	O=C(O)CC1=C(C(C2=CC=C(OC)C=C2)N3C4=C(C=CC=C4)SC3=N1)C(OC)=O		O=C(O)CC1=C(C(/C=C/C2=CC=CC=C2)NC(N1)=O)C(OC)=O
DB13	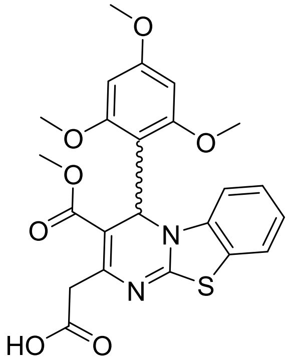	DU25	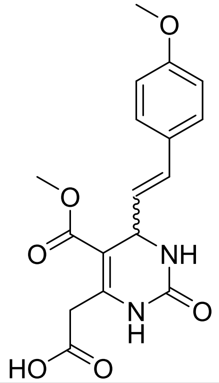
	O=C(O)CC1=C(C(C2=C(OC)C=C(OC)C=C2OC)N3C4=C(C=CC=C4)SC3=N1)C(OC)=O		O=C(O)CC1=C(C(/C=C/C2=CC=C(OC)C=C2)NC(N1)=O)C(OC)=O
DB14	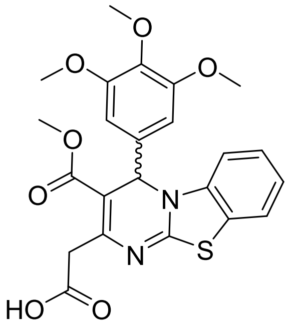	DU26	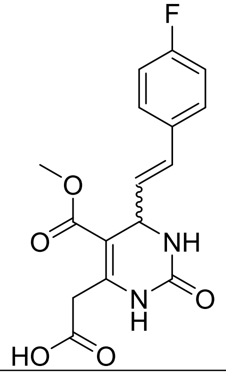
	O=C(O)CC1=C(C(C2=CC(OC)=C(OC)C(OC)=C2)N3C4=C(C=CC=C4)SC3=N1)C(OC)=O		O=C(O)CC1=C(C(/C=C/C2=CC=C(F)C=C2)NC(N1)=O)C(OC)=O
DB15	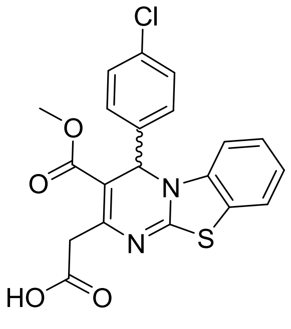	DU27	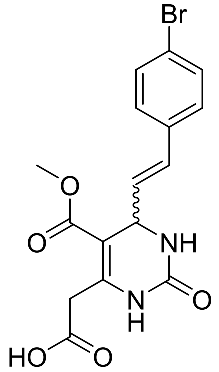
	O=C(O)CC1=C(C(C2=CC=C(Cl)C=C2)N3C4=C(C=CC=C4)SC3=N1)C(OC)=O		O=C(O)CC1=C(C(/C=C/C2=CC=C(Br)C=C2)NC(N1)=O)C(OC)=O

DB16	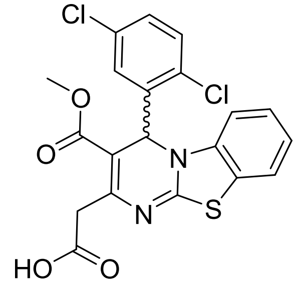	DU28	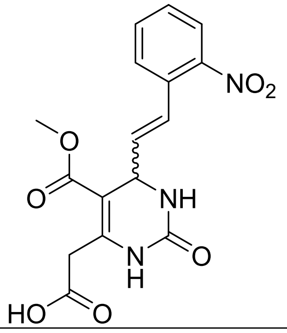
	O=C(O)CC1=C(C(C2=C(Cl)C=CC(Cl)=C2)N3C4=C(C=CC=C4)SC3=N1)C(OC)=O		O=C(O)CC1=C(C(/C=C/C2=C([N+]([O-])=O)C=CC=C2)NC(N1)=O)C(OC)=O
DB17	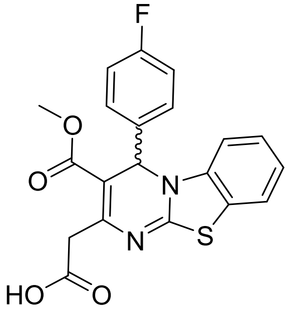	DU29	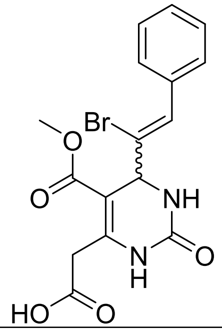
	O=C(O)CC1=C(C(C2=CC=C(F)C=C2)N3C4=C(C=CC=C4)SC3=N1)C(OC)=O		O=C(O)CC1=C(C(/C(Br)=C/C2=CC=CC=C2)NC(N1)=O)C(OC)=O
DB18	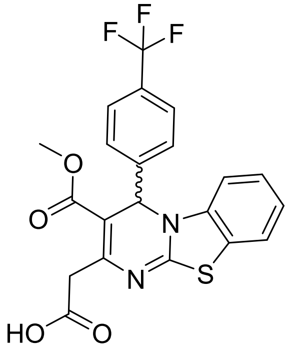	DU30	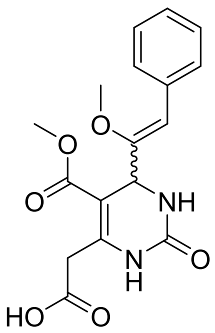
	O=C(O)CC1=C(C(C2=CC=C(C(F)(F)F)C=C2)N3C4=C(C=CC=C4)SC3=N1)C(OC)=O		O=C(O)CC1=C(C(/C(OC)=C/C2=CC=CC=C2)NC(N1)=O)C(OC)=O
DB19	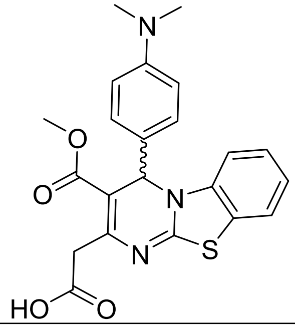	DZ01	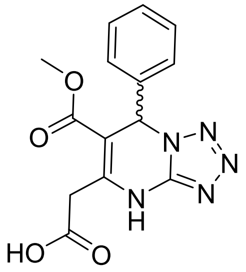
	O=C(O)CC1=C(C(C2=CC=C(N(C)C)C=C2)N3C4=C(C=CC=C4)SC3=N1)C(OC)=O		O=C(OC)C1=C(CC(O)=O)NC2=NN=NN2C1C3=CC=CC=C3

DB20	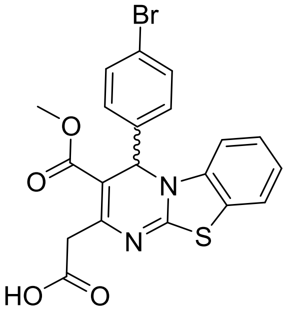	DZ02	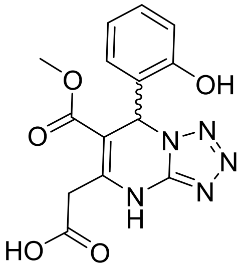
	O=C(O)CC1=C(C(C2=CC=C(Br)C=C2)N3C4=C(C=CC=C4)SC3=N1)C(OC)=O		O=C(OC)C1=C(CC(O)=O)NC2=NN=NN2C1C3=C(O)C=CC=C3
DB21	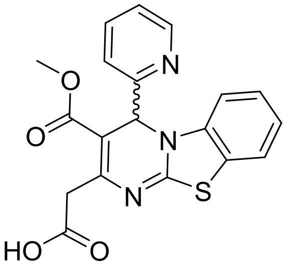	DZ03	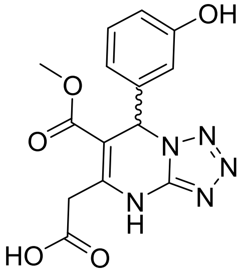
	O=C(O)CC1=C(C(C2=NC=CC=C2)N3C4=C(C=CC=C4)SC3=N1)C(OC)=O		O=C(OC)C1=C(CC(O)=O)NC2=NN=NN2C1C3=CC(O)=CC=C3
DB22	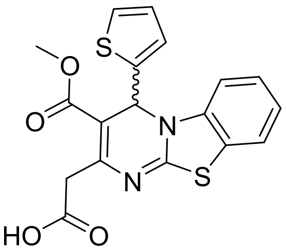	DZ04	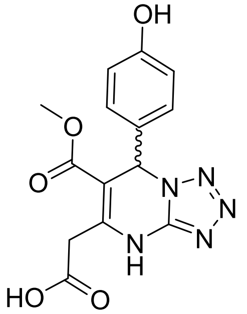
	O=C(O)CC1=C(C(C2=CC=CS2)N3C4=C(C=CC=C4)SC3=N1)C(OC)=O		O=C(OC)C1=C(CC(O)=O)NC2=NN=NN2C1C3=CC=C(O)C=C3
DB23	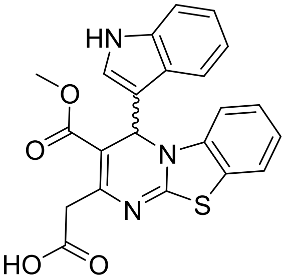	DZ05	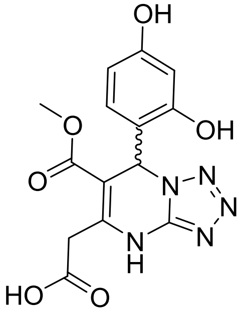
	O=C(O)CC1=C(C(C2=CNC3=C2C=CC=C3)N4C5=C(C=CC=C5)SC4=N1)C(OC)=O		O=C(OC)C1=C(CC(O)=O)NC2=NN=NN2C1C3=C(O)C=C(O)C=C3

DB24	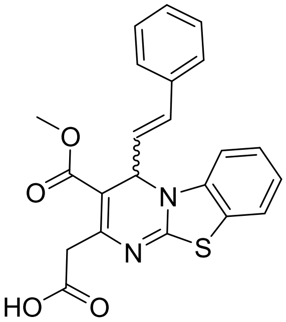	DZ06	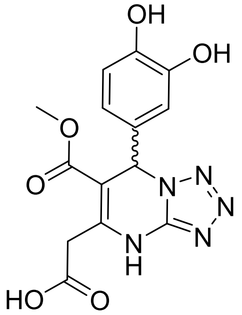
	O=C(O)CC1=C(C(/C=C/C2=CC=CC=C2)N3C4=C(C=CC=C4)SC3=N1)C(OC)=O		O=C(OC)C1=C(CC(O)=O)NC2=NN=NN2C1C3=CC(O)=C(O)C=C3
DB25	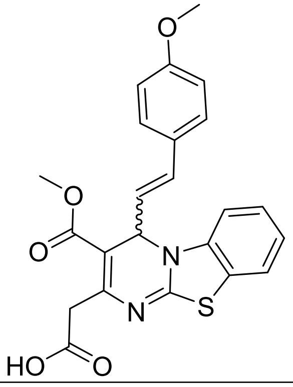	DZ07	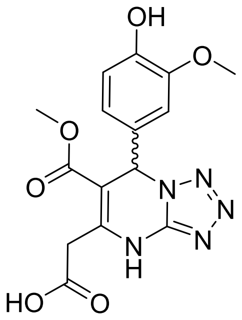
	O=C(O)CC1=C(C(/C=C/C2=CC=C(OC)C=C2)N3C4=C(C=CC=C4)SC3=N1)C(OC)=O		O=C(OC)C1=C(CC(O)=O)NC2=NN=NN2C1C3=CC(OC)=C(O)C=C3
DB26	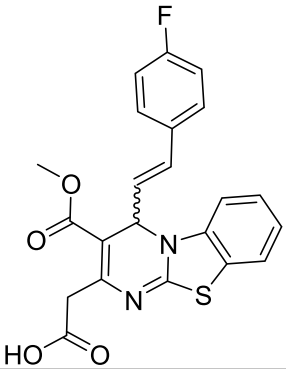	DZ08	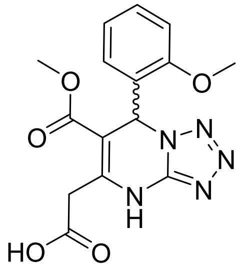
	O=C(O)CC1=C(C(/C=C/C2=CC=C(F)C=C2)N3C4=C(C=CC=C4)SC3=N1)C(OC)=O		O=C(OC)C1=C(CC(O)=O)NC2=NN=NN2C1C3=C(OC)C=CC=C3
DB27	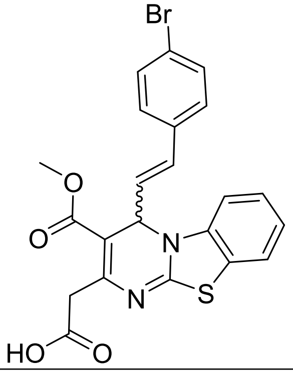	DZ09	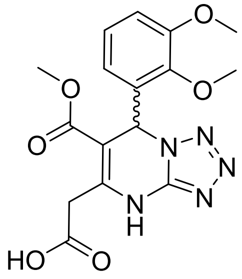
	O=C(O)CC1=C(C(/C=C/C2=CC=C(Br)C=C2)N3C4=C(C=CC=C4)SC3=N1)C(OC)=O		O=C(OC)C1=C(CC(O)=O)NC2=NN=NN2C1C3=C(OC)C(OC)=CC=C3

DB28	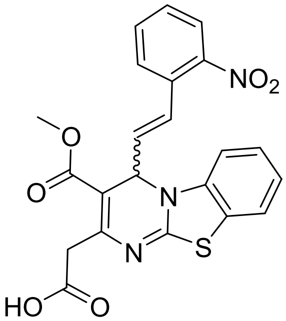	DZ10	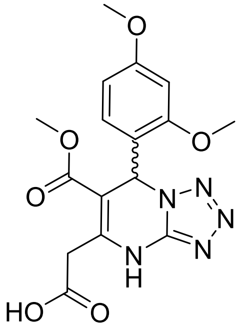
	O=C(O)CC1=C(C(/C=C/C2=C([N+]([O-])=O)C=CC=C2)N3C4=C(C=CC=C4)SC3=N1)C(OC)=O		O=C(OC)C1=C(CC(O)=O)NC2=NN=NN2C1C3=C(OC)C=C(OC)C=C3
DB29	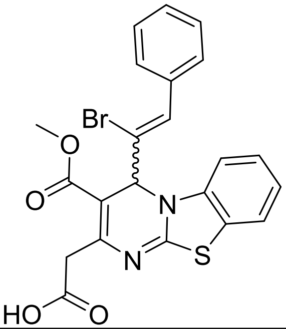	DZ11	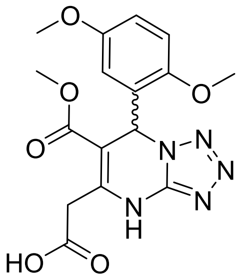
	O=C(O)CC1=C(C(/C(Br)=C/C2=CC=CC=C2)N3C4=C(C=CC=C4)SC3=N1)C(OC)=O		O=C(OC)C1=C(CC(O)=O)NC2=NN=NN2C1C3=C(OC)C=CC(OC)=C3
DB30	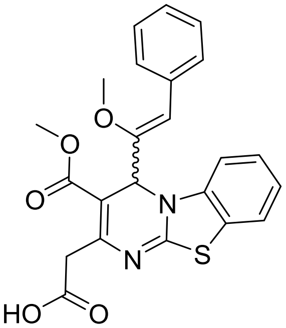	DZ12	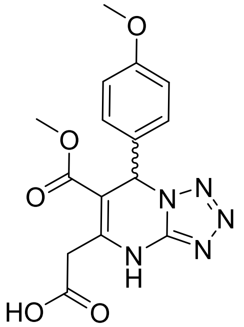
	O=C(O)CC1=C(C(/C(OC)=C/C2=CC=CC=C2)N3C4=C(C=CC=C4)SC3=N1)C(OC)=O		O=C(OC)C1=C(CC(O)=O)NC2=NN=NN2C1C3=CC=C(OC)C=C3
DT01	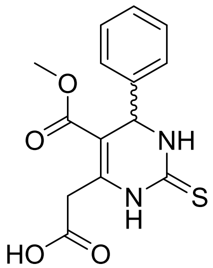	DZ13	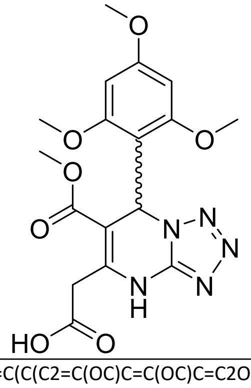
	O=C(O)CC1=C(C(C2=CC=CC=C2)NC(N1)=S)C(OC)=O		O=C(O)CC1=C(C(C2=C(OC)C=C(OC)C=C2OC)N3C(N1)=NN=N3)C(OC)=O

DT02	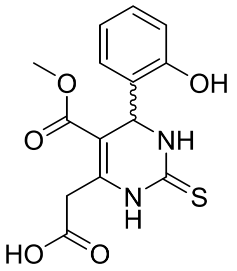	DZ14	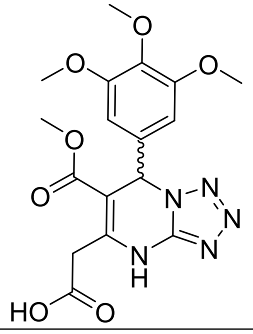
	O=C(O)CC1=C(C(C2=C(O)C=CC=C2)NC(N1)=S)C(OC)=O		O=C(OC)C1=C(CC(O)=O)NC2=NN=NN2C1C3=CC(OC)= C(OC)C(OC)=C3
DT03	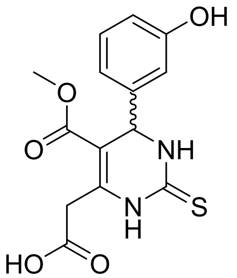	DZ15	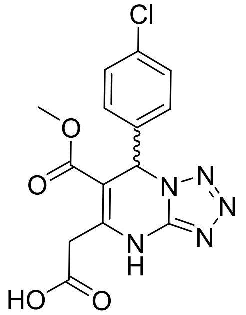
	O=C(O)CC1=C(C(C2=CC(O)=CC=C2)NC(N1)=S)C(OC)=O		O=C(OC)C1=C(CC(O)=O)NC2=NN=NN2C1C3=CC=C(Cl) C=C3
DT04	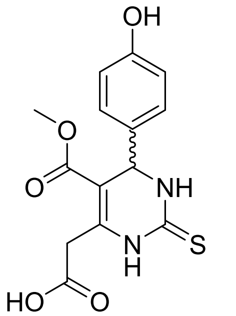	DZ16	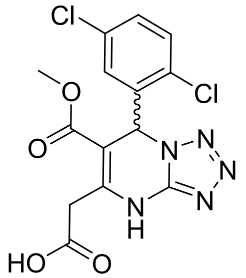
	O=C(O)CC1=C(C(C2=CC=C(O)C=C2)NC(N1)=S)C(OC)=O		O=C(OC)C1=C(CC(O)=O)NC2=NN=NN2C1C3=C(Cl)C=C C(Cl)=C3
DT05	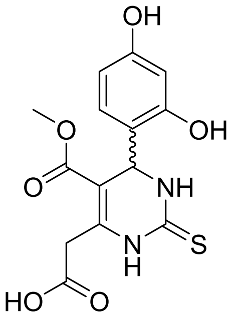	DZ17	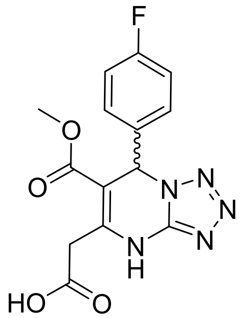
	O=C(O)CC1=C(C(C2=CC=C(O)C=C2)NC(N1)=S)C(OC)=O		O=C(OC)C1=C(CC(O)=O)NC2=NN=NN2C1C3=CC=C(F) C=C3

DT06	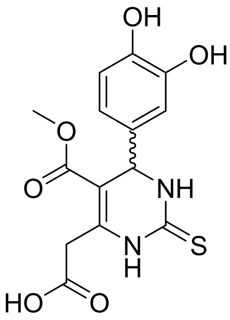	DZ18	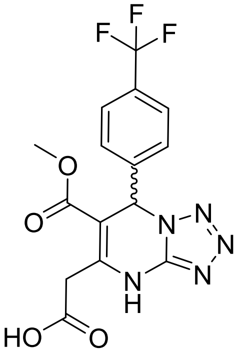
	O=C(O)CC1=C(C(C2=CC(O)=C(O)C=C2)NC(N1)=S)C(OC)=O		O=C(OC)C1=C(CC(O)=O)NC2=NN=NN2C1C3=CC=C(C(F)(F)F)C=C3
DT07	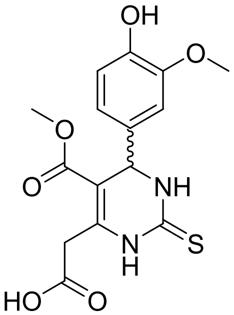	DZ19	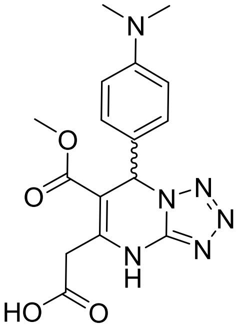
	O=C(O)CC1=C(C(C2=CC(OC)=C(O)C=C2)NC(N1)=S)C(OC)=O		O=C(OC)C1=C(CC(O)=O)NC2=NN=NN2C1C3=CC=C(N(C)C)C=C3
DT08	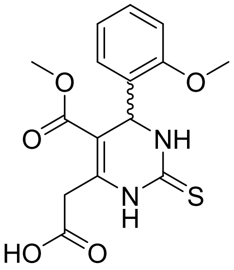	DZ20	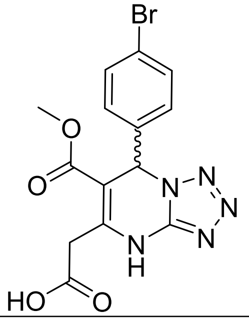
	O=C(O)CC1=C(C(C2=C(OC)C=CC=C2)NC(N1)=S)C(OC)=O		O=C(OC)C1=C(CC(O)=O)NC2=NN=NN2C1C3=CC=C(Br)C=C3
DT09	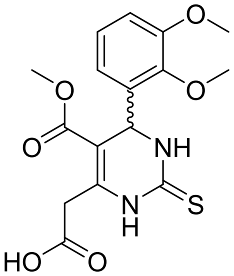	DZ21	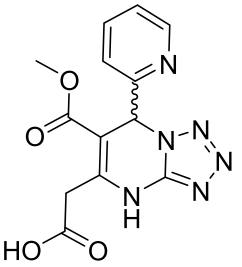
	O=C(O)CC1=C(C(C2=C(OC)C(OC)=CC=C2)NC(N1)=S)C(OC)=O		O=C(O)CC1=C(C(C2=NC=CC=C2)N3C(N1)=NN=N3)C(OC)=O

DT10	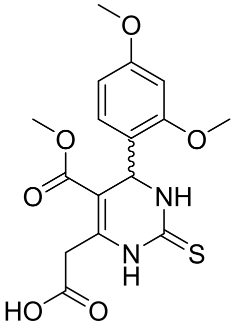	DZ22	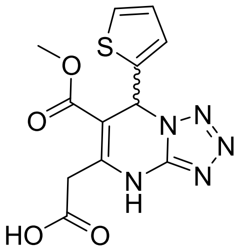
	O=C(O)CC1=C(C(C2=C(OC)C=C(OC)C=C2)NC(N1)=S)C(OC)=O		O=C(O)CC1=C(C(C2=CC=CS2)N3C(N1)=NN=N3)C(OC)=O
DT11	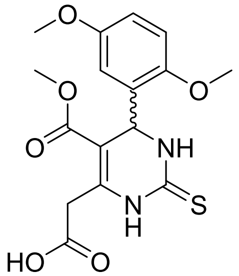	DZ23	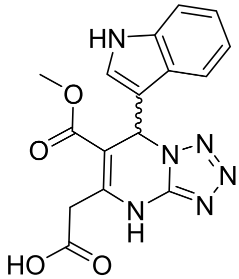
	O=C(O)CC1=C(C(C2=C(OC)C=CC(OC)=C2)NC(N1)=S)C(OC)=O		O=C(O)CC1=C(C(C2=CNC3=C2C=CC=C3)N4C(N1)=NN=N4)C(OC)=O
DT12	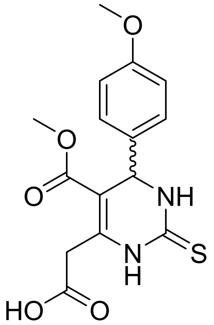	DZ24	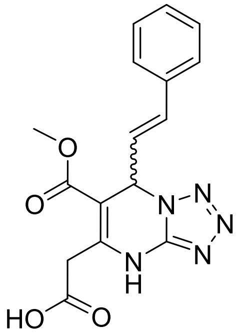
	O=C(O)CC1=C(C(C2=CC=C(OC)C=C2)NC(N1)=S)C(OC)=O		O=C(O)CC1=C(C(/C=C/C2=CC=CC=C2)N3N=NN=C3N1)C(OC)=O
DT13	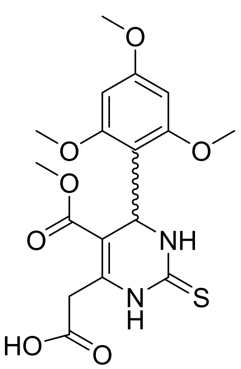	DZ25	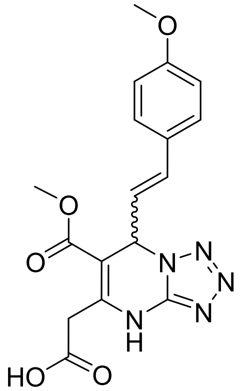
	O=C(O)CC1=C(C(C2=C(OC)C=C(OC)C=C2OC)NC(N1)=S)C(OC)=O		O=C(O)CC1=C(C(/C=C/C2=CC=C(OC)C=C2)N3N=NN=C3N1)C(OC)=O

DT14	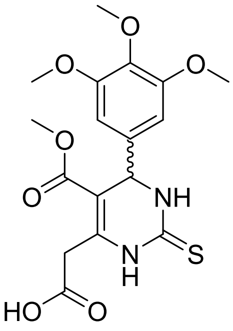	DZ26	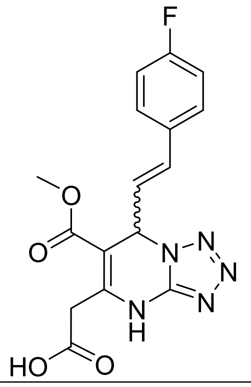
	O=C(O)CC1=C(C(C2=CC(OC)=C(OC)C(OC)=C2)NC(N1)=S)C(OC)=O		O=C(O)CC1=C(C(/C=C/C2=CC=C(F)C=C2)N3N=NN=C3N1)C(OC)=O
DT15	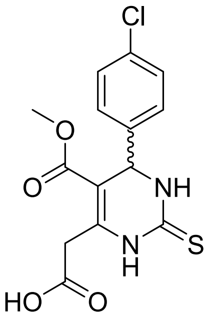	DZ27	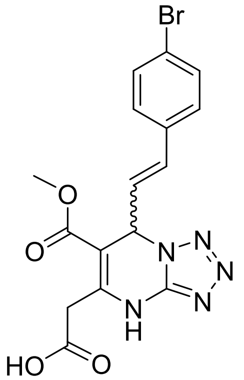
	O=C(O)CC1=C(C(C2=CC=C(Cl)C=C2)NC(N1)=S)C(OC)=O		O=C(O)CC1=C(C(/C=C/C2=CC=C(Br)C=C2)N3N=NN=C3N1)C(OC)=O
DT16	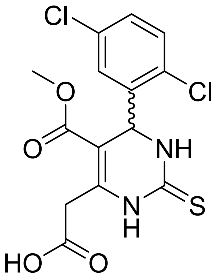	DZ28	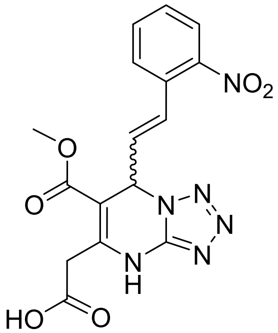
	O=C(O)CC1=C(C(C2=C(Cl)C=CC(Cl)=C2)NC(N1)=S)C(OC)=O		O=C(O)CC1=C(C(/C=C/C2=C([N+]([O-])=O)C=CC=C2)N3N=NN=C3N1)C(OC)=O
DT17	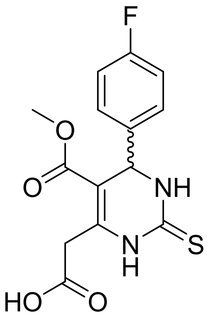	DZ29	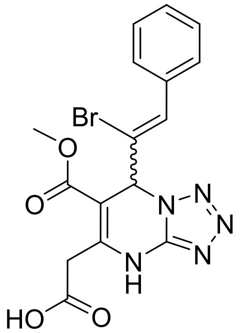
	O=C(O)CC1=C(C(C2=CC=C(F)C=C2)NC(N1)=S)C(OC)=O		O=C(O)CC1=C(C(/C(Br)=C/C2=CC=CC=C2)N3N=NN=C3N1)C(OC)=O

DT18	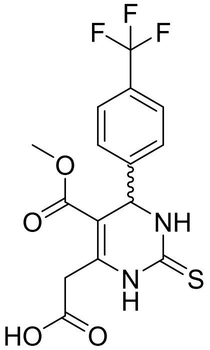	DZ30	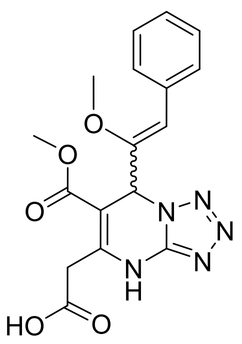
	O=C(O)CC1=C(C(C2=CC=C(C(F)(F)F)C=C2)NC(N1)=S)C(OC)=O		O=C(O)CC1=C(C(/C(OC)=C/C2=CC=CC=C2)N3N=NN=C3N1)C(OC)=O
DT19	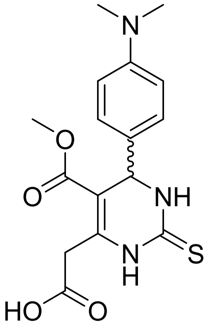	DBA2	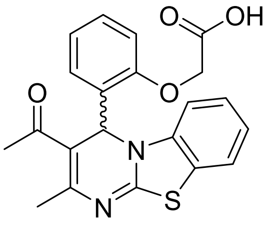
	O=C(O)CC1=C(C(C2=CC=C(N(C)C)C=C2)NC(N1)=S)C(OC)=O		OC(COC1=C(C=CC=C1)C2C(C(C)=O)=C(N=C3N2C4=C(S3)C=CC=C4)C)=O
DT20	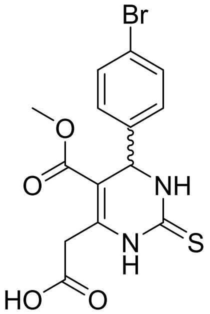	DBA3	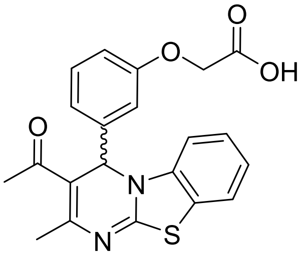
	O=C(O)CC1=C(C(C2=CC=C(Br)C=C2)NC(N1)=S)C(OC)=O		CC(N=C1N2C3=C(S1)C=CC=C3)=C(C2C4=CC(OCC(O)=O)=CC=C4)C(C)=O
DT21	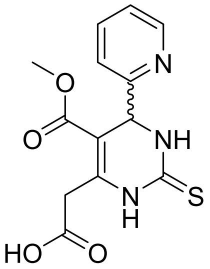	DBA4	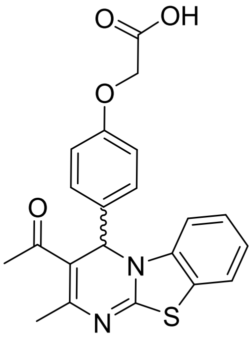
	O=C(O)CC1=C(C(C2=NC=CC=C2)NC(N1)=S)C(OC)=O		CC(N=C1N2C3=C(S1)C=CC=C3)=C(C2C4=CC=C(C=C4)OCC(O)=O)C(C)=O

DT22	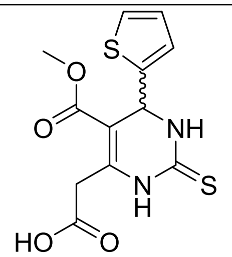	DBE2	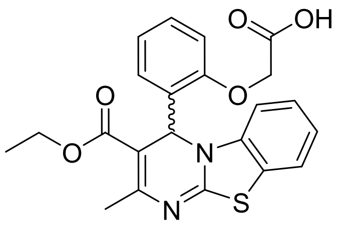
	O=C(O)CC1=C(C(C2=CC=CS2)NC(N1)=S)C(OC)=O		OC(COC1=C(C=CC=C1)C2C(C(OCC)=O)=C(N=C3N2C4=C(S3)C=CC=C4)C)=O
DT23	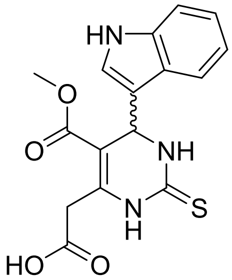	DBE3	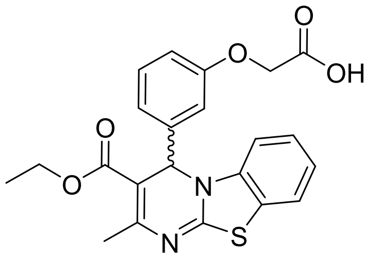
	O=C(O)CC1=C(C(C2=CNC3=C2C=CC=C3)NC(N1)=S)C(OC)=O		CC(N=C1N2C3=C(S1)C=CC=C3)=C(C2C4=CC(OCC(O)=O)=CC=C4)C(OCC)=O
DT24	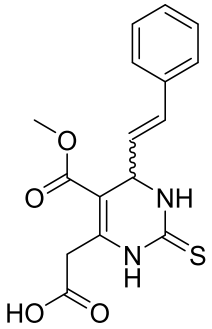	DBE4	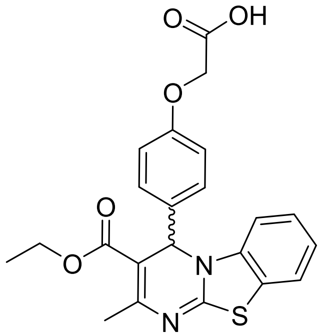
	O=C(O)CC1=C(C(/C=C/C2=CC=CC=C2)NC(N1)=S)C(OC)=O		CC(N=C1N2C3=C(S1)C=CC=C3)=C(C2C4=CC=C(C=C4)OCC(O)=O)C(OCC)=O
DT25	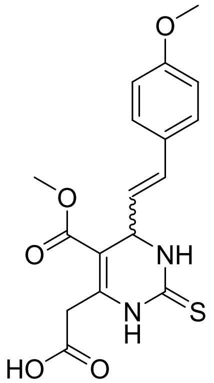	DTA2	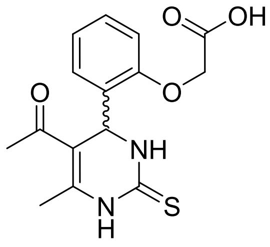
	O=C(O)CC1=C(C(/C=C/C2=CC=C(OC)C=C2)NC(N1)=S)C(OC)=O		OC(COC1=C(C=CC=C1)C2C(C(C)=O)=C(NC(N2)=S)C)=O

DT26	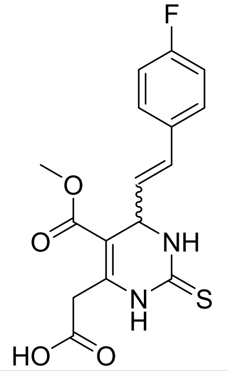	DTA3	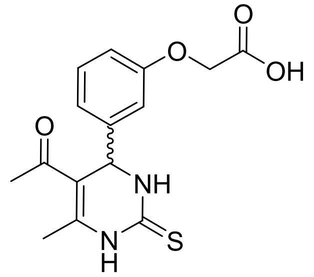
	O=C(O)CC1=C(C(/C=C/C2=CC=C(F)C=C2)NC(N1)=S)C(OC)=O		CC(NC(NC1C2=CC(OCC(O)=O)=CC=C2)=S)=C1C(C)=O
DT27	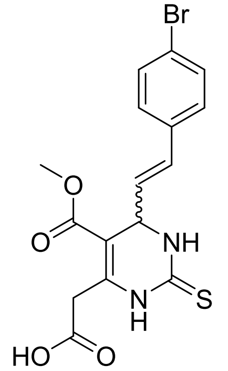	DTA4	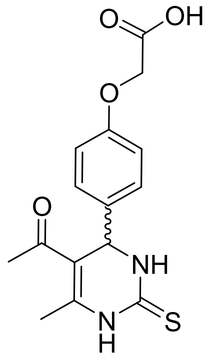
	O=C(O)CC1=C(C(/C=C/C2=CC=C(Br)C=C2)NC(N1)=S)C(OC)=O		CC(NC(NC1C2=CC=C(C=C2)OCC(O)=O)=S)=C1C(C)=O
DT28	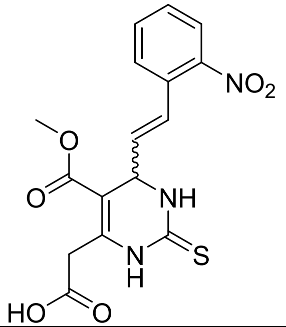	DTE2	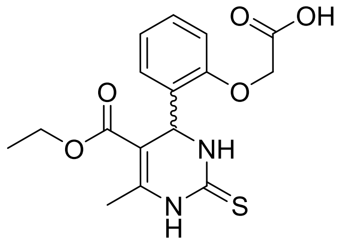
	O=C(O)CC1=C(C(/C=C/C2=C([N+]([O])=O)C=CC=C2)NC(N1)=S)C(OC)=O		OC(COC1=C(C=CC=C1)C2C(C(OCC)=O)=C(NC(N2)=S)C)=O
DT29	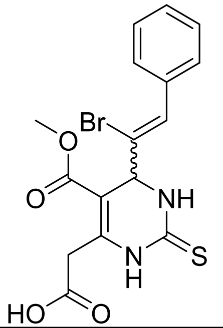	DTE3	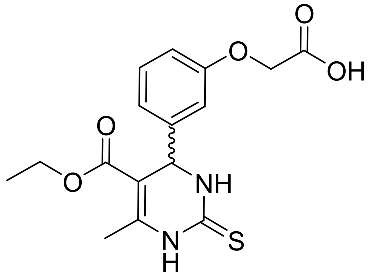
	O=C(O)CC1=C(C(/C(Br)=C/C2=CC=CC=C2)NC(N1)=S)C(OC)=O		CC(NC(NC1C2=CC(OCC(O)=O)=CC=C2)=S)=C1C(OCC)=O

DT30	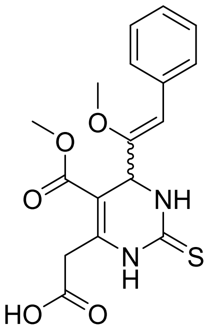	DTE4	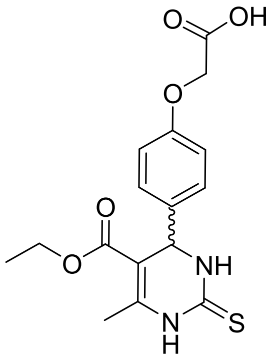
	O=C(O)CC1=C(C(/C(OC)=C/C2=CC=CC=C2)NC(N1)=S)C(OC)=O		CC(NC(NC1C2=CC=C(C=C2)OCC(O)=O)=S)=C1C(OCC)=O
DU01	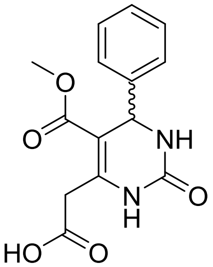	DUA2	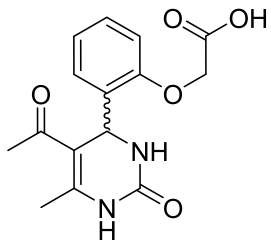
	O=C(O)CC1=C(C(C2=CC=CC=C2)NC(N1)=O)C(OC)=O		OC(COC1=C(C=CC=C1)C2C(C(C)=O)=C(NC(N2)=O)C)=O
DU02	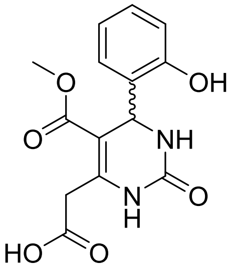	DUA3	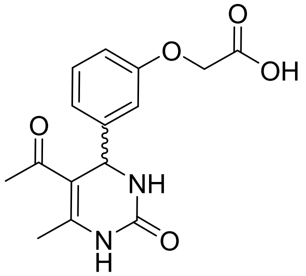
	O=C(O)CC1=C(C(C2=C(O)C=CC=C2)NC(N1)=O)C(OC)=O		CC(NC(NC1C2=CC(OCC(O)=O)=CC=C2)=O)=C1C(C)=O
DU03	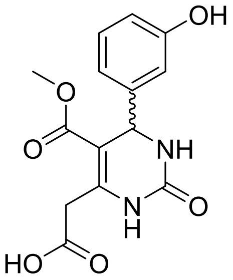	DUA4	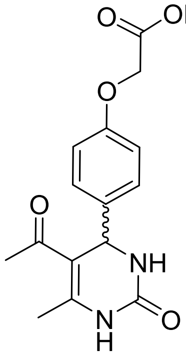
	O=C(O)CC1=C(C(C2=CC(O)=CC=C2)NC(N1)=O)C(OC)=O		CC(NC(NC1C2=CC=C(C=C2)OCC(O)=O)=O)=C1C(C)=O

DU04	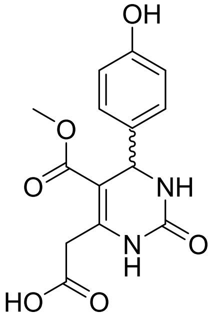	DUE2	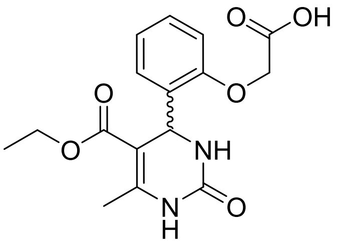
	O=C(O)CC1=C(C(C2=CC=C(O)C=C2)NC(N1)=O)C(OC)=O		OC(COC1=C(C=CC=C1)C2C(C(OCC)=O)=C(NC(N2)=O)C)=O
DU05	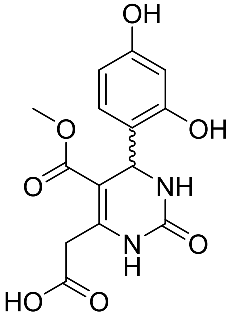	DUE3	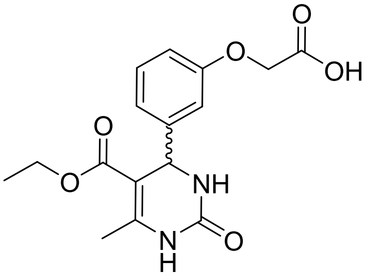
	O=C(O)CC1=C(C(C2=C(O)C=C(O)C=C2)NC(N1)=O)C(OC)=O		CC(NC(NC1C2=CC(OCC(O)=O)=CC=C2)=O)=C1C(OCC)=O
DU06	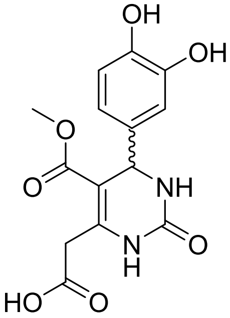	DUE4	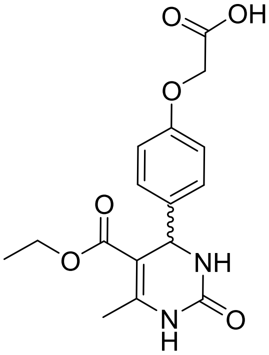
	O=C(O)CC1=C(C(C2=CC(O)=C(O)C=C2)NC(N1)=O)C(OC)=O		CC(NC(NC1C2=CC=C(C=C2)OCC(O)=O)=O)=C1C(OCC)=O
DU07	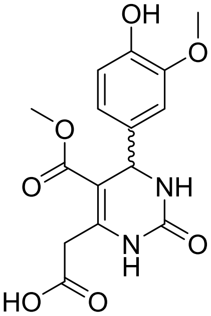	DZA2	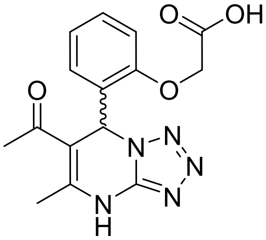
	O=C(O)CC1=C(C(C2=CC(OC)=C(O)C=C2)NC(N1)=O)C(OC)=O		OC(COC1=C(C=CC=C1)C2C(C(C)=O)=C(NC3=NN=NN32)C)=O

DU08	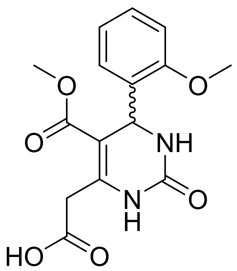	DZA3	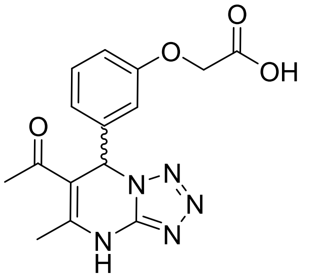
	O=C(O)CC1=C(C(C2=C(OC)C=CC=C2)NC(N1)=O)C(OC)=O		CC(NC1=NN=NN1C2C3=CC(OCC(O)=O)=CC=C3)=C2C(C)=O
DU09	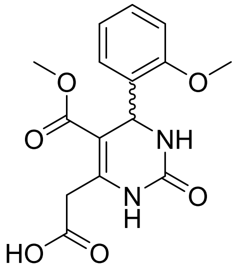	DZA4	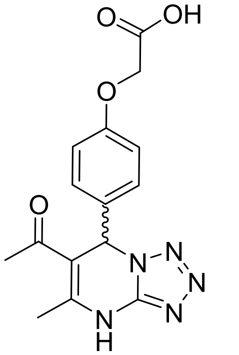
	O=C(O)CC1=C(C(C2=C(OC)C=CC=C2)NC(N1)=O)C(OC)=O		CC(NC1=NN=NN1C2C3=CC=C(C=C3)OCC(O)=O)=C2C(C)=O
DU10	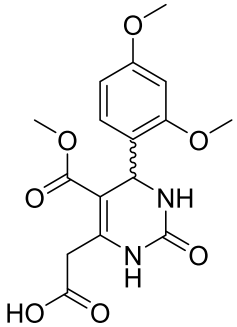	DZE2	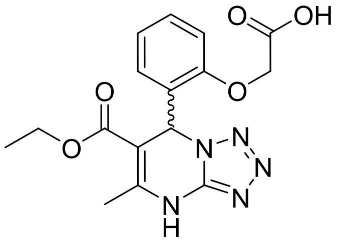
	O=C(O)CC1=C(C(C2=C(OC)C=C(OC)C=C2)NC(N1)=O)C(OC)=O		OC(COC1=C(C=CC=C1)C2C(C(OCC)=O)=C(NC3=NN=NN32)C)=O
DU11	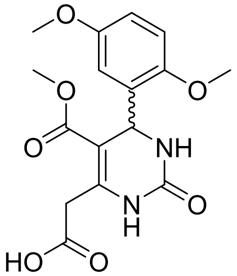	DZE3	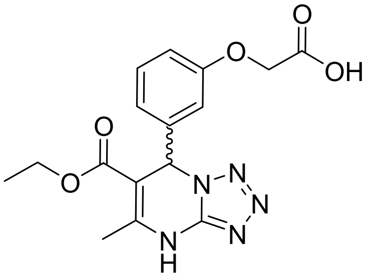
	O=C(O)CC1=C(C(C2=C(OC)C=CC(OC)=C2)NC(N1)=O)C(OC)=O		CC(NC1=NN=NN1C2C3=CC(OCC(O)=O)=CC=C3)=C2C(OCC)=O

DU12	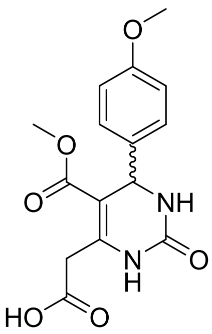	DZE4	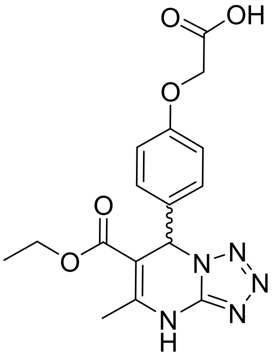
	O=C(O)CC1=C(C(C2=CC=C(OC)C=C2)NC(N1)=O)C(OC)=O		CC(NC1=NN=NN1C2C3=CC=C(C=C3)OCC(O)=O)=C2C(OCC)=O

Table S2. List of marketed antiinflammatory drugs which used as references

No.	Compound (CAS)	SMILES	Docking score (kcal/mol)
1	Phenylbutazone (50-33-9)	CCCCC1C(=O)N(N(C1=O)C1CCCCC1)C1CCCCC1	-42.74
2	Difenamizole (20170-20-1)	CN(C(C(=O)NC1CC(NN1C1CCCCC1)C1CCCCC1)C)C	-49.36
3	Sulfinpyrazone (57-96-5)	O=C1N(C2CCCCC2)N(C(=O)C1CCS(=O)C1CCCCC1)C1CCCCC1	79.08
4	Aspirin (50-78-2)	CC(=O)OC1CCCCC1C(=O)O	-42.00
5	Salsalate (552-94-3)	OC1CCCCC1C(=O)OC1CCCCC1C(=O)O	-54.68
6	Benorilate (5003-48-5)	CC(=O)NC1CCC(CC1)OC(=O)C1CCCCC1OC(=O)C	-52.35
7	Diclofenac (15307-86-5)	OC(=O)CC1CCCCC1NC1C(Cl)CCCC1Cl	-62.16
8	Sulindac (38194-50-2)	OC(=O)CC1=C(C)C(=CC2CCC(CC2)S(=O)C)C2C1CC(F)CC2	-27.48
9	Indomethacin (53-86-1)	COC1CCC2C(C1)C(CC(=O)O)C(N2C(=O)C1CCC(CC1)Cl)C	-40.52
10	Mofezolac (78967-07-4)	COC1CCC(CC1)C1C(ONC1C1CCC(CC1)OC)CC(=O)O	-66.84
11	Bromfenac (91714-94-2)	OC(=O)CC1CCCC(C1N)C(=O)C1CCC(CC1)Br	-60.38
12	Etodolac (41340-25-4)	CCC1(OCCC2C1[NH]C1C2CCCC1CC)CC(=O)O	-26.38
13	Piroxicam (36322-90-4)	O=C(C1=C(O)C2CCCCC2S(=O)(=O)N1C)NC1CCCCN1	-34.46
14	Meloxicam (71125-38-7)	CC1CNC(S1)NC(=O)C1=C(O)C2CCCCC2S(=O)(=O)N1C	-27.21
15	Ibuprofen (15687-27-1)	CC(CC1CCC(CC1)C(C(=O)O)C)C	-47.69
16	Flurbiprofen (5104-49-4)	OC(=O)C(C1CCC(C(C1)F)C1CCCCC1)C	-50.33
17	Naproxen (22204-53-1)	COC1CCC2C(C1)CCC(C2)C(C(=O)O)C	-53.80
18	Ketoprofen (22071-15-4)	OC(=O)C(C1CCCC(C1)C(=O)C1CCCCC1)C	-58.12
19	Fenoprofen (29679-58-1)	OC(=O)C(C1CCCC(C1)OC1CCCCC1)C	-53.34
20	Oxaprozin (22204-53-1)	OC(=O)CCC1NC(C(O1)C1CCCCC1)C1CCCCC1	-60.69
21	Mefenamic acid (61-68-7)	OC(=O)C1CCCCC1NC1CCCC(C1C)C	-56.69
22	Azapropazone (13539-59-8)	CCCC1C(=O)N2N(C1=O)C1CC(C)CCC1NC2N(C)C	-55.82
23	Etofenamate (30544-47-9)	OCCOCCOC(=O)C1CCCCC1NC1CCCC(C1)C(F)(F)F	-68.04
24	Celecoxib (169590-42-5)	CC1CCC(CC1)C1CC(NN1C1CCC(CC1)S(=O)(=O)N)C(F)(F)F	-63.75
25	Robenacoxib (220991-32-2)	CCC1CCC(C(C1)CC(=O)O)NC1C(F)C(F)CC(C1F)F	-64.80
26	Firocoxib (189954-96-9)	O=C1OC(C(=C1OCC1CC1)C1CCC(CC1)S(=O)(=O)C)(C)C	-58.82
27	Nimesulide (51803-78-2)	[O-][N+](=O)C1CCC(C(C1)OC1CCCCC1)NS(=O)(=O)C	-56.53
28	Nabumetone (42924-53-8)	COC1CCC2C(C1)CCC(C2)CCC(=O)C	-50.27
29	Benzydamine (642-72-8)	CN(CCCOC1NN(C2C1CCCC2)CC1CCCCC1)C	-56.44
30	Lumiracoxib (220991-20-8)	OC(=O)CC1CC(C)CCC1NC1C(F)CCCC1Cl	-64.16

Table S3 The type of contribution for each residue based on decomposition energy (in kcal/mol)

Residue	van der Waals	Electrostatic	Polar Solvation	Non-Polar Solvation	Total	Type of contribution
			**COX-2:LUR**			
Tyr317	-0.793	-2.850	2.816	-0.017	-0.843	van der Waals
Val318	-1.816	-0.418	0.563	-0.197	-1.868	van der Waals
Leu321	-1.287	-1.330	1.188	-0.123	-1.552	van der Waals
Ser322	-0.842	-1.338	1.507	-0.090	-0.763	van der Waals
Tyr354	-0.612	-7.789	4.635	-0.069	-3.835	Electrostatic
Phe487	-0.777	-0.724	1.169	-0.055	-0.387	van der Waals
Val492	-1.539	0.895	-0.727	-0.146	-1.516	van der Waals
Gly495	-0.895	0.066	-0.509	-0.062	-1.400	van der Waals
Ala496	-1.852	0.685	-0.575	-0.176	-1.918	van der Waals
Ser499	0.335	-10.566	5.477	-0.099	-4.853	Electrostatic
			**COX-2:RDUE2**			
Tyr317	-0.582	-2.822	2.696	-0.016	-0.724	van der Waals
Val318	-1.541	-0.501	0.530	-0.217	-1.728	van der Waals
Leu321	-2.340	-0.184	1.132	-0.171	-1.562	van der Waals
Ser322	-1.693	-1.221	1.798	-0.171	-1.287	van der Waals
Tyr354	0.203	-8.986	4.955	-0.087	-3.915	Electrostatic
Phe487	-1.803	-0.753	1.291	-0.119	-1.385	van der Waals
Val492	-2.044	1.385	-0.955	-0.200	-1.815	van der Waals
Gly495	-0.733	-1.156	0.613	-0.034	-1.311	van der Waals
Ala496	-2.210	-0.063	0.362	-0.307	-2.218	van der Waals
Ser499	0.210	-10.069	5.688	-0.106	-4.278	Electrostatic
			**COX-2:SDT29**			
Tyr317	-0.510	-2.682	2.493	-0.016	-0.715	van der Waals
Val318	-1.292	-0.287	0.331	-0.194	-1.442	van der Waals
Leu321	-1.685	-0.890	1.103	-0.154	-1.626	van der Waals
Ser322	-1.284	-1.343	1.801	-0.156	-0.982	van der Waals
Tyr354	0.368	-8.263	4.230	-0.086	-3.751	Electrostatic
Phe487	-0.714	-0.508	0.937	-0.052	-0.338	van der Waals
Val492	-2.826	-1.304	0.822	-0.286	-3.593	van der Waals & Electrostatic
Gly495	-0.977	0.161	0.189	-0.056	-0.683	van der Waals
Ala496	-1.604	1.381	-1.177	-0.127	-1.526	van der Waals
Ser499	-0.486	-8.220	4.984	-0.165	-3.888	Electrostatic

Table S4 Analysis of hydrogen bonds between ligands and residues.

Acceptor	Donor	Fraction (%)	Average distance (Å)	Average angle (º)
**COX-2:LUR**				
LUR (O3)	Ser499 (OG)	93.7	2.60	165.54
LUR (O1)	Tyr354 (OH)	88.5	2.72	161.92
**COX-2:RDUE2**				
RDUE2 (O6)	Tyr354 (OH)	77.6	2.67	166.21
RDUE2 (O1)	Ser499 (OG)	64.3	2.69	164.90
**COX-2:SDT29**				
SDT29 (O3)	Tyr354 (OH)	98.7	2.65	165.49
SDT29 (O4)	Ser499 (OG)	96.7	2.67	164.16
Val492 (O)	SDT29 (N1)	26.5	2.88	152.13

Table S5 Analysis of van der Waals interaction between ligands and residues.

Ligand	Residue	Fraction (%)	Average distance (Å)
**COX-2:LUR**			
LUR (O3)	Ser499 (CB)	93.7	3.25
LUR (C2)	Ser499 (OG)	63.3	3.38
LUR (C4)	Tyr354 (OH)	53.4	3.38
LUR (O1)	Ser499 (OG)	21.1	3.35
**COX-2:RDUE2**			
RDUE2 (O5)	Leu321 (O)	71.9	3.23
RDUE2 (C12)	Leu321 (O)	66.1	3.21
RDUE2 (O1)	Ser499 (CB)	57.4	3.34
RDUE2 (C14)	Val492 (O)	52.2	3.35
RDUE2 (C14)	Met491 (O)	29.4	3.39
RDUE2 (O2)	Leu321 (CD1)	23.5	3.37
RDUE2 (C13)	Val492 (O)	12.6	3.40
RDUE2 (N2)	Leu321 (CB)	5.85	3.43
**COX-2:SDT29**			
SDT29 (C15)	Ser499 (OG)	71.3	3.32
SDT29 (O1)	Val318 (CG1)	67.2	3.28
SDT29 (C9)	Tyr324 (OH)	57.6	3.22
SDT29 (C14)	Ser499 (OG)	48.3	3.34
SDT29 (C4)	Val492 (O)	41.3	3.35
SDT29 (C8)	Tyr324 (OH)	37.1	3.29
SDT29 (C6)	Val492 (O)	34.4	3.32
SDT29 (S1)	Leu353 (CD2)	3.05	3.42
